# The Caspase-1-EGR4 axis regulates macrophage repolarization in acute myeloid leukemia cells

**DOI:** 10.1038/s41598-026-41381-x

**Published:** 2026-02-27

**Authors:** Yi Qian, Yue Chen, Zu-Xi Feng, Xiao-Feng Zhu, Li Zhang, Hao Xiong, Xiang-Hui Zhang, Jun Bai, Yan-Hong Li, Yu-Xian Wang, Li-Juan Li, Lian-Sheng Zhang

**Affiliations:** 1 Department of Hematology, The Second Hospital and Clinical Medical School, Lanzhou University, Lanzhou, 730030 China; 2 Department of Hematology, The Affiliated Hospital of Southwest Medical University, Luzhou, 646000 China; 3 Key Laboratory of the Hematology of Gansu Province, The Second Hospital and Clinical Medical School, Lanzhou University, Lanzhou, 730030 China; 4 Gansu Clinical Medical Research Center of Hematology (National Sub-Center), The Second Hospital and Clinical Medical School, Lanzhou University, Lanzhou, 730030 China; 5 The First People’s Hospital of Tianshui, Tianshui, 741000 China

**Keywords:** Acute myeloid leukemia, CASP1, EGR4, Macrophage polarization, Tumor microenvironment, Cancer, Cell biology, Computational biology and bioinformatics, Immunology, Oncology

## Abstract

The polarization of tumor-associated macrophages (TAMs) toward an M2-like phenotype critically promotes acute myeloid leukemia (AML) progression. Building on the clinical observation that Caspase-1 (CASP1) expression is elevated in AML and correlates with M2 macrophage abundance, we identify a novel signaling axis in AML cells, involving CASP1 and the transcription factor early growth response protein 4 (EGR4), that orchestrates macrophage polarization. Knockdown (KD) of CASP1 in human AML cells (THP-1, MOLM-13) shifted their secretome, which consequently skewed macrophage polarization away from the M2 phenotype at multiple levels. Mechanistically, transcriptomic sequencing revealed that CASP1 KD significantly upregulated EGR4 expression. Crucially, EGR4 interference partially reversed the macrophage-polarizing effects of CASP1 KD, establishing EGR4 as an essential downstream effector. In a xenograft model using NOD/SCID mice—a defined system for studying human AML-macrophage crosstalk—CASP1 KD potently suppressed tumor growth. Immunohistochemical analysis revealed a remodeled microenvironment characterized by reduced proliferation (Ki67), upregulated EGR4, suppression of the M2-associated IL-10/p-STAT3 pathway and CD206, alongside a concomitant increase in M1-associated marker CD86. In conclusion, our integrated analysis delineates a novel AML cell-intrinsic pathway wherein CASP1 represses EGR4, thereby enabling an M2-like macrophage phenotype via the IL-10/p-STAT3 pathway. The identification of this CASP1-EGR4 axis identifies it as a promising therapeutic target for reshaping the immunosuppressive microenvironment in AML.

**Supplementary Information:**

The online version contains supplementary material available at 10.1038/s41598-026-41381-x.

## Introduction

Characterized by significant heterogeneity, acute myeloid leukemia (AML) is a hematologic neoplasm whose progression depends on both intrinsic leukemic cell activity and extrinsic immunomodulation within the tumor microenvironment (TME)^1–3^. The immunosuppressive TME is a cornerstone of AML progression and therapy resistance^4,5^. Within the TME, tumor-associated macrophages (TAMs) that adopt an M2 phenotype are pivotal drivers of immunosuppression and leukemic cell survival^6,7^. Reprogramming these M2-like TAMs to an anti-tumoral M1 state represents a promising therapeutic strategy; however, the key molecular drivers that orchestrate this polarization in AML remain incompletely understood.

Caspase-1 (CASP1), a cysteine protease best known for its role in inflammasome signaling and pyroptosis, has been implicated in various cancers^8^. Its function in cancer, however, remains context-dependent and paradoxical, exhibiting both tumor-suppressive and tumor-promoting effects^9^. This functional duality implies potential roles beyond cell-intrinsic inflammasome signaling, possibly involving immunomodulatory crosstalk within the TME. Studies have found that CASP1 can promote TAMs differentiation by cleaving PPARγ, thereby driving tumor progression^10^. Building on these dual roles and the established link between inflammatory signaling and the immunosuppressive niche, we hypothesized that the upregulation of CASP1 observed in AML might be a key driver in shaping a pro-tumor microenvironment, potentially through the modulation of macrophage polarization. The mechanistic basis for such a non-canonical, immune-modulatory function of CASP1 in AML, however, remained entirely unknown.

Here, we hypothesize that CASP1 in AML cells contributes to an immunosuppressive microenvironment by orchestrating macrophage polarization towards an M2 phenotype. To test this, we employed a conditioned medium (CM)-based approach to model tumor cell-macrophage communication, combined with transcriptomic profiling and in vivo xenograft experiments. Our integrated analysis identified the early growth response protein 4 (EGR4) as a key downstream node whose expression is negatively regulated by CASP1 in AML cells. Furthermore, we delineated a functional axis wherein CASP1-mediated downregulation of EGR4 activates the IL-10/p-STAT3 pathway, thereby promoting M2 macrophage polarization. Our work therefore not only provides mechanistic insight into the pro-tumoral function of CASP1 but also proposes a mechanistic framework centered on the CASP1-EGR4 functional axis as a novel and therapeutically targetable node for reprogramming the immunosuppressive microenvironment in AML.

## Materials and methods

### Ethical statement

#### Cell lines

This study used commercially available, well-characterized human leukemia cell lines (THP-1 and MOLM-13). The use of such established cell lines is in accordance with standard research practice and institutional guidelines.

### Animal ethics approval

All procedures involving animals were approved by The Animal Ethics Committee of The Second Affiliated Hospital of Lanzhou University (Approval Ref: D2025-732) and were conducted in strict accordance with relevant national and institutional guidelines. This study is reported in accordance with the ARRIVE guidelines.

### Generation of stable CASP1-knockdown AML cell lines via lentiviral transduction

The human AML cell lines THP-1 and MOLM-13 were purchased from Procell Life Science and Technology Co., Ltd (Wuhan, China). All cell lines were maintained at 37 °C with 5% CO₂ in RPMI-1640 medium (Gibco, USA) supplemented with 10% fetal bovine serum (FBS; PAN-Biotech, Germany, Cat# ST30-3302). Cells were routinely passaged when the cell density reached 1 × 10⁶ cells/mL to maintain exponential growth. Lentiviral vectors (GV493) carrying either a short hairpin RNA (shRNA) targeting CASP1 (shCASP1) or a non-targeting control (shNC) were obtained from GeneChem (Shanghai, China). These vectors were based on the hU6-MCS-CBh-gcGFP-IRES-puromycin backbone. Subsequently, AML cells were transduced with the resulting viral particles in the absence of transfection reagents. To establish stable, polyclonal knockdown pools, transduced cells were subjected to a 6-day selection with puromycin to eliminate non-transduced cells. Following selection, transduction efficiency was confirmed by assessing the GFP-positive cell population via fluorescence microscopy and flow cytometry. We evaluated the KD efficiency by assessing CASP1 expression at both the mRNA and protein levels, using using RT-qPCR and western blot, respectively. The successful establishment of CASP1 KD, which was evaluated using both RT-qPCR for mRNA and western blot for protein analysis, was a prerequisite for subsequent experiments. Three distinct shRNAs targeting CASP1 (pGMLV-shCASP1) were screened. From three tested shRNAs targeting CASP1 (shCASP1#1: 5′-CACCACTGAAAGAGTGACTTT-3′; shCASP1#2: 5′-TTGGAAGACTCATTGAACATA-3′; shCASP1#3: 5′-TTTCTTGGAGACATCCCACAA-3′), shCASP1#2 was chosen for subsequent experiments due to its highest KD efficiency. The non-targeting control shRNA (shNC) sequence was: 5′-TTCTCCGAACGTGTCACGT-3′.

### siRNA transfection

shCASP1 cells were transfected at ~ 70% confluence with siEGR4 or negative control siRNA (siNC; Genepharma) using GP-transfect-Mate reagent. The medium was refreshed after 6–8 h, and CM was harvested 72 h post-transfection for subsequent experiments. The siRNA sequences were: siEGR4, sense 5′-GCUUCUUCAUUCAGGCAGUTT-3′, antisense 5′-ACUGCCUGAAUGAAGAAGCTT-3′.

### Preparation of conditioned medium

AML cells were subjected to combined genetic manipulation: lentiviral transduction with shNC or shCASP1, followed by transfection with siNC or siEGR4 using GP-transfect-Mate reagent. The final groups were shNC, shCASP1, and shCASP1 + siEGR4. Following a 48-hour culture, the supernatant was harvested and subjected to successive centrifugation steps at increasing g-forces: 300 × g for 5 min to remove cells, and then 1,000 × g for 15 min to pellet cellular debris. To generate the conditioned medium (CM) for subsequent experiments, this clarified fraction was combined with fresh complete medium at a 3:1 ratio^11,12^.

### Induction, polarization, and phenotypic evaluation of macrophages from THP-1 cells

To generate unpolarized M0 macrophages, THP-1 cells were treated with 150 ng/mL phorbol 12-myristate 13-acetate (PMA; AbMole, China) for 48 h^13,14^. These M0 macrophages were subsequently incubated with conditioned media (CM) from shNC, shCASP1, shNC+siNC, or shCASP1 + siEGR4 cells for 48–72 h to induce polarization. The resulting macrophage phenotypes were evaluated using a combination of methods: **Quantitative gene and cellular analyses**: The mRNA levels of M1-related genes (IL-6, TNF-α, IL-1β, and IL-12) and M2-related genes (TGF-β, Arg1, IL-10, and IL-4) were quantified by RT-qPCR. The percentages of CD163⁺ and CD86⁺ cells within the populations were determined by flow cytometry. **Protein-level assessments**: Changes in the protein expression of CD206 (M2-associated marker) and CD86 (M1-associated marker) were evaluated by western blot, with representative blot images presented. Additionally, CD206 expression patterns were visualized by immunofluorescence, with representative micrographs shown to illustrate observed differences across groups.

### Comparative transcriptomic analysis of shCASP1 and shNC cells

The contracted service with Shanghai Majorbio Bio-pharm Technology Co., Ltd. covered the full workflow from RNA extraction of Trizol-homogenized samples (shCASP1 and shNC AML cells, 5 × 10⁶ cells per replicate, *n* = 3) through library preparation and sequencing to final bioinformatic data analysis.

### RNA extraction and RT-qPCR analysis

Total RNA was first isolated using Trizol (Servicebio, China); subsequently, this RNA was converted to cDNA employing the SweScript SuperMix kit (Servicebio, China, G3337-100) for subsequent quantitative PCR. A ThermoFisher 7500 system was employed to run the qPCR reactions with SYBR Green Master Mix (Servicebio, China, G3321-05). Gene expression was quantified after normalization to β-actin via the 2^(–ΔΔCT) method (see Supplementary Table S1 for primer sequences).

### Western blot analysis

Protein extracts obtained from cells lysed in inhibitor-supplemented (protease and phosphatase) RIPA buffer were subjected to SDS-PAGE. After electrophoretic transfer to PVDF membranes, blocking was performed prior to an overnight incubation at 4 °C with the primary antibodies of interest. Post-wash, membranes were incubated with HRP-linked secondary antibodies. For normalization purposes, the enhanced chemiluminescence signals were quantified against the levels of GAPDH or β-actin after detection. All antibodies used for western blot 、flow cytometry、immunofluorescence and Immunohistochemistry were specific for human antigens. Supplementary Table S2 contains detailed information on all antibodies used.

### Cell immunofluorescence and imaging: Fixation

We processed cells on coverslips for fixation by incubation with 4% paraformaldehyde (15 min, room temperature). **Washing**: Following this fixation step, we rinsed the coverslips thoroughly with phosphate-buffered saline (PBS). **Permeabilization and blocking**: A permeabilization/blocking solution (0.3% Triton X-100, 5% BSA) was applied to the samples for 1 h to achieve cell permeabilization and reduce nonspecific binding. **Primary antibody incubation**: Cells were labeled with an anti-CD206 antibody (1:300; Invitrogen, MA5-32498) overnight at 4 °C. **Secondary antibody staining / labeling**: To detect bound primary antibodies, samples were incubated in the dark for 1 h at room temperature with a 1:500 dilution of CoraLite^®^ Plus 594–conjugated goat anti-rabbit secondary antibody (Proteintech, RGAR004). **Nuclear staining**: We stained the nuclei using DAPI. **Mounting**: We then mounted the coverslips with an anti-fade medium. We captured the images with a ZEISS LSM 880 confocal microscope.

### Flow cytometry assay

Flow cytometric evaluation of CD163 and CD86 cell surface expression was performed. The cells were first resuspended in ice-cold PBS. They were then stained for 40 min at 4 °C in the dark using PE-conjugated antibodies specific for CD163 or CD86. Following a wash step, data were acquired on a BD FACSLyric flow cytometer and analyzed using Kaluza Analysis Software (Beckman Coulter, v2.1).

#### Gating strategy

A sequential gating strategy was employed to identify single, viable cells. First, the main cell population was identified on an FSC-A versus SSC-A dot plot, excluding events with low scatter signals (debris). Subsequently, single cells were selected from this population using an FSC-H versus FSC-A plot, where a tight gate was applied along the diagonal to exclude cell doublets and aggregates.

#### Data analysis

The fluorescence intensity of the unstained control was used to set the negative boundary for the PE channel. The threshold for a positive signal was defined so that > 99% of events in the unstained control fell within the negative gate. The percentage of CD163^+^ or CD86^+^ cells was then quantified from the pre-gated population of single, viable cells.

### In vivo xenograft model

Male NOD/SCID mice (SPF, 4–6 weeks old, 18–20 g; Jiangsu Huachuang Xinnuo Pharmaceutical Technology Co., Ltd) were housed under specific pathogen-free conditions of the Medical Experiment Center of Lanzhou University. Mice were randomly allocated into two groups: shCASP1 and shNC (*n* = 4 per group). To establish tumors, 5 × 10⁶ MOLM-13 cells were inoculated subcutaneously into the right axilla as previously described^15,16^. Tumor growth was monitored regularly. On day 26, mice received an intratumoral injection of 1 × 10⁷ THP-1-derived M0 macrophages. All mice were euthanized on day 30. For euthanasia, mice were first anesthetized with isoflurane (5% for induction) and then administered a lethal dose of sodium pentobarbital (100 mg/kg, delivered as a 2% solution). Death was confirmed by the absence of respiratory and cardiac arrest, followed by cervical dislocation. Excised tumors were photographed, weighed, and fixed in 4% paraformaldehyde for subsequent processing (dehydration, paraffin embedding, sectioning) and immunohistochemical analysis.

### Hematoxylin-eosin staining

The tissue sections underwent standard histological processing: deparaffinization, rehydration through a graded ethanol series, and final hematoxylin-eosin (HE) staining. Mounting of the dehydrated and cleared sections was performed with neutral balsam to enable subsequent microscopic analysis.

### Immunohistochemical staining and evaluation

For IHC analysis, consecutive sections were processed through deparaffinization, rehydration, and antigen retrieval. After blocking, samples were probed with specific primary antibodies in a cold room (4 °C) overnight. Detection was carried out using HRP-conjugated secondary antibodies and a DAB substrate, with hematoxylin applied as a nuclear counterstain before final mounting. Quantification of IHC staining: To quantify IHC staining, we employed the semi-quantitative Immunoreactive Score (IRS)^17,18^. IHC staining was evaluated semiquantitatively using a dual-parameter scoring system. The intensity score was defined as: 0 (none, unstained), 1 (weak, light/pale yellow), 2 (moderate, brownish yellow), and 3 (strong, tan). The proportion score was: 0 (< 4%), 1 (4–10%), 2 (11–50%), 3 (51–80%), and 4 (> 81%) positive cells. Calculation of the final IRS for each case involved multiplying the intensity score by the percentage score. This evaluation was conducted across five randomly selected fields, with each field containing approximately 100 cells. Two independent, blinded pathologists performed all evaluations.

### Statistical analysis

All statistical analyses and the corresponding graphs were generated using GraphPad Prism 10. Results are expressed as the mean ± standard deviation, following a minimum of three independent experiments. Depending on the data distribution and the number of groups being compared, statistical analyses were conducted with either an unpaired t-test or one-way ANOVA. Asterisks indicate the following levels of statistical significance : *p < 0.05; **p < 0.01; ***p < 0.001; ****p < 0.0001.

### Note on macrophage polarization markers

To comprehensively assess macrophage polarization, we employed a panel of well-established markers optimized for different detection techniques. For flow cytometric analysis at the single-cell level, we used surface CD163 and CD86 as canonical markers for M2-like and M1-like phenotypes, respectively^19^. For protein-level quantification by western blot and spatial visualization by immunofluorescence, we utilized CD206 (another widely used M2 marker) alongside CD86^20^. The use of both CD163 and CD206, which are distinct but commonly co-expressed markers associated with the M2 polarization state, strengthens our assessment of the M2 phenotype across multiple experimental modalities.

## Results

Building on earlier observations linking elevated CASP1 expression to adverse clinical outcomes and M2 macrophage enrichment in AML, this work sought to elucidate the mechanistic basis of this association. We examined the direct impact of AML cell–intrinsic CASP1 loss on macrophage reprogramming. Treatment with conditioned medium from CASP1-KD AML cells consistently downregulated M2-associated markers and upregulated the M1-associated markers in macrophages, a shift validated across transcriptional, translational, and cellular assays. Subsequent investigation identified the transcription factor EGR4 as a key downstream target of CASP1, a pathway that suppresses IL-10/p-STAT3 signaling. In vivo, CASP1 KD not only attenuated AML progression but also reprogrammed the TAM population, characterized by a loss of M2-like features. Collectively, these findings define the CASP1-EGR4 axis as a pivotal regulator within AML cells that orchestrates an immunosuppressive TME.

### Generation of stable CASP1 knockdown models in THP-1 and MOLM-13 cells

**Fig. 1:**
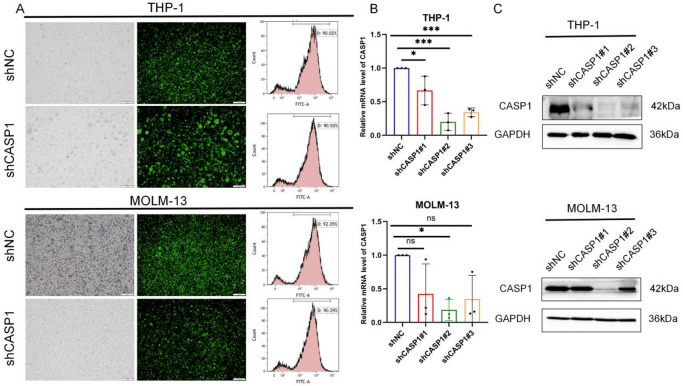
Efficient CASP1 KD in THP-1 and MOLM-13 cells. (**A**) (left) GFP fluorescence and (right) FCM analysis of GFP expression levels in the stable polyclonal cell pools. (**B**,**C**) Evaluation of CASP1-KD efficiency by qPCR and western blot. Values represent mean ± SD of triplicate experiments. Statistical significance versus the shNC group is shown, with *, ***, and ns representing *p* < 0.05, *p* < 0.001, and not significant, respectively. Cropped blot images are shown. Groupings of lanes from non-adjacent parts of the same gel are indicated by black borders. Full-length, uncropped original blots are provided in Supplementary Dataset.

Selection of the THP-1 and MOLM-13 cell lines from a screened human AML panel was based on their pronounced endogenous CASP1 expression. Stable CASP1-KD polyclonal pools were generated in these cells via lentiviral transduction of three independent shRNAs (shCASP1#1, shCASP1#2, shCASP1#3), with a non-targeting shRNA (shNC) serving as control. High transduction efficiency was confirmed by ubiquitous GFP fluorescence and flow cytometry (Fig. 1A). Based on qPCR analysis, shCASP1#2 was selected for all further studies as it achieved approximately 80% KD of CASP1 mRNA in both THP-1 and MOLM-13 cell lines (Fig. 1B), a result supported by western blot (Fig. 1C).

#### CASP1 knockdown in AML cells alters macrophage polarization at the transcriptional level

**Fig. 2:**
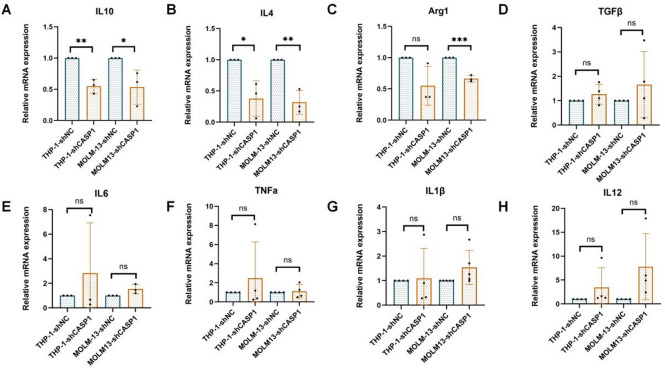
CASP1 KD in AML cells shifts macrophages away from an M2 phenotype at the transcriptional level. qPCR analysis of (**A**–**D**) M2 and (**E**–**H**) M1 macrophage polarization markers after treatment with conditioned medium from shCASP1 or shNC cells. Data are mean ± SD (*n* ≥ 3). *p < 0.05, ** *p* < 0.01, *** *p* < 0.001, ns (not significant) vs. shNC group.

To assess the paracrine effect of AML-cell-intrinsic CASP1 on macrophage polarization, we treated M0 macrophages with CM from shCASP1 or control (shNC) AML cells. RT-qPCR results showed a marked downregulation of key M2-associated genes following treatment with shCASP1 CM (Fig. 2). While *IL-10* and *IL-4* were markedly downregulated in both THP-1 and MOLM-13 models, *Arg1* expression was significantly reduced in MOLM-13-derived CM and showed a consistent decreasing trend, albeit non-significant, in THP-1 cells. The observed increase in the expression of M1-related genes *(IL-6*, *TNF-α*, *IL-1β*, and *IL-12*) failed to achieve statistical significance. *TGF-β* expression remained unaltered. These findings indicate that CASP1 KD in AML cells primarily attenuates the M2 polarization program in macrophages.

#### Protein-level validation of macrophage polarization shift

**Fig. 3:**
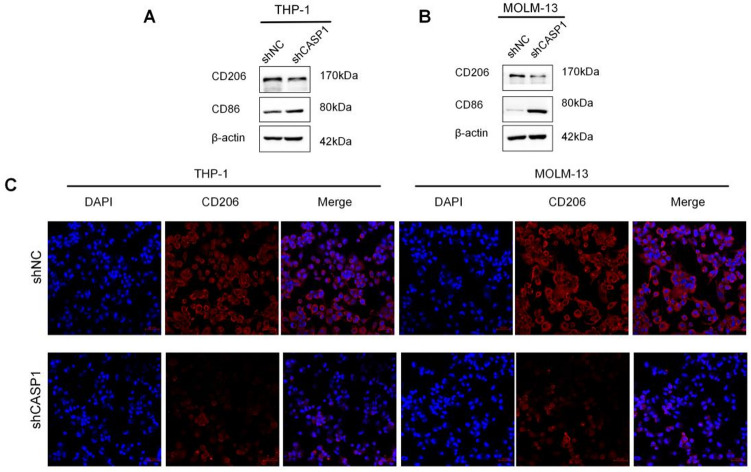
CASP1 KD in AML cells alters macrophage polarization at the protein level. (**A**,**B**) Representative western blot results showing the expression of M1 (CD86) and M2 (CD206)**-**associated markers in macrophages treated with conditioned medium (CM) from shCASP1 or shNC AML cells. Visual assessment shows decreased CD206 and increased CD86 levels in the shCASP1 group. (**C**) Representative IF images of CD206 (red) in macrophages. Following staining with DAPI (blue), cell nuclei were identified. Compared with the shNC group, the shCASP1 group displayed a marked reduction in both CD206 fluorescence intensity and the number of CD206-positive cells. Scale bar, 50 μm. Cropped blot images are shown. Groupings of lanes from non-adjacent parts of the same gel are indicated by black borders. Full-length, uncropped original blots are provided in Supplementary Dataset. Quantitative analysis of the M1 marker CD86 by flow cytometry is presented in (Fig. 4).

Validation of the transcriptional findings was extended through analysis of macrophage polarization at the protein level. Compared to the shNC control, shCASP1-CM treatment significantly decreased the M2-associated marker CD206 and concomitantly increased the M1-associated marker CD86^20^, as shown by western blot(Fig. 3A, B). The observed reduction in both CD206 fluorescence intensity and the CD206^+^ cell count by IF staining supports the conclusion of M2 phenotype suppression in the shCASP1 group (Fig. 3C), aligning with the preceding data. Collectively, the western blot and immunofluorescence data indicate that the secretome from CASP1-KD AML cells is associated with a reduction in M2-associated markers (CD206) and an increase in M1-associated marker (CD86) at the protein level.

#### Flow cytometry validates the macrophage polarization shift at the cellular level

**Fig. 4:**
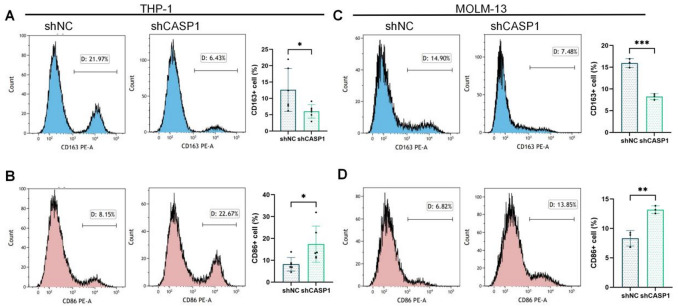
Flow cytometric analysis of macrophage polarization markers. The THP-1 (**A**,**B**) and MOLM-13 (**C**,**D**) models are presented in each panel with representative histograms (left) and quantitative data (right). (**A**,**C**) CD163^+^ M2 macrophages; (**B**,**D**) CD86^+^ M1 macrophages. Values shown are mean ± SD (*n* ≥ 3 independent experiments). *, **, ***, or ns denotes *p* < 0.05, *p* < 0.01, *p* < 0.001,or not significant versus the shNC control, respectively.

We next assessed polarization at the single-cell level by FCM. CASP1 KD consistently and significantly reduced the proportion of CD163^+^ M2 macrophages^19,21^ across both AML models(Fig. 4A, C). In contrast, shCASP1-CM markedly increased the percentage of CD86^+^ M1 macrophages in the MOLM-13 model (Fig. 4D), a trend that was also statistically significant in the THP-1 model (Fig. 4B). Collectively, these data indicate that CASP1 KD effectively suppresses M2 polarization, with a concomitant upregulation of the M1-associated marker CD86 (*P* < 0.05 in THP-1; *P* < 0.01 in MOLM-13) in our AML cell models.

### CASP1 KD upregulates the transcription factor EGR4 in AML cells

**Fig. 5:**
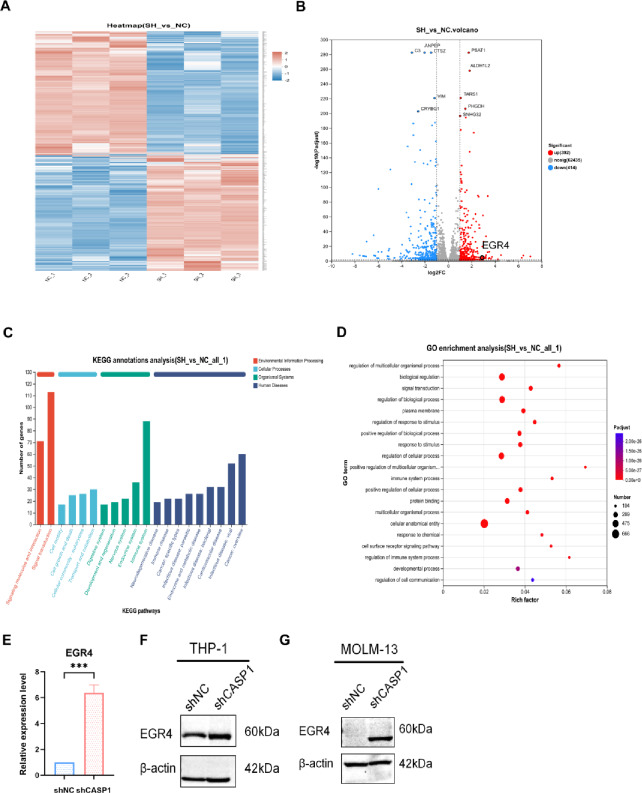
Transcriptomic profiling identifies EGR4 as a key transcriptional target upregulated by CASP1 KD in AML cells. (**A**) Heat plot of the global transcriptome from shNC and shCASP1 THP-1 cells. (**B**) Distribution of DEGs is shown in a volcano plot, where statistically significant upregulations and downregulations are highlighted in red and blue, respectively. (**C**) KEGG pathway (Kanehisa Laboratories, https://www.kegg.jp/) classification of the DEGs, showing the most enriched functional categories. (**D**) GO (The Gene Ontology Resource, http://geneontology.org/) enrichment analysis for biological processes, showing the most significantly enriched terms. (**E**) qPCR validation of *EGR4* mRNA expression in THP-1 cells from the indicated groups. (**F**,**G**) EGR4 protein expression was assessed by western blot in THP-1 and MOLM-13 cells. We calculated the data from three independent experiments as the mean ± SD, using ***p < 0.001 as the threshold for statistical significance compared to the shNC group. Cropped blot images are shown. Groupings of lanes from non-adjacent parts of the same gel are indicated by black borders. Full-length, uncropped original blots are provided in Supplementary Dataset.

mRNA sequencing was performed on shCASP1 and shNC THP-1 cells to elucidate how CASP1 reprograms the microenvironment. Unsupervised clustering and volcano plot analysis confirmed distinct transcriptomic profiles and numerous differentially expressed genes (DEGs) in shCASP1 cells (Fig. 5A, B). Significant enrichment of DEGs in immune-related pathways and processes—including signal transduction and immune system regulation—was identified by KEGG^22^ and Gene Ontology (GO) analyses (Fig. 5C, D). To identify key downstream mediators responsible for the altered secretome, we adopted a candidate-based approach grounded in our transcriptomic data. From the DEG list, we pre-selected a panel of candidate genes for validation based on the magnitude of expression change (fold-change) and their reported relevance to immune regulation or macrophage function in the literature. The candidates included both up-regulated (e.g., *IL7R*, *SERPINE1*, *STEAP4*, *EGR4*) and down-regulated genes (e.g., *FCN1*, *MMP12*, *CCL8*, *CXCL14*). Subsequent RT-qPCR validation in THP-1 cells revealed that EGR4 exhibited the most consistent and statistically significant upregulation upon CASP1 KD (Fig. 5E), aligning perfectly with the sequencing data. The expression changes of several other candidates were either non-significant, inconsistent with the sequencing trend, or less statistically robust compared to EGR4, leading us to prioritize EGR4 as the lead candidate for further mechanistic investigation. This transcriptional upregulation identified in THP-1 cells was further confirmed at the protein level by western blot in both THP-1 and MOLM-13 cell lines (Fig. 5F, G). Thus, through a combination of transcriptomic screening in THP-1 cells and cross-cell-line protein validation, we identify EGR4 as a key downstream transcriptional regulator modulated by CASP1 in AML cells.

### EGR4 KD reverses the macrophage polarization shift induced by CASP1 KD

**Fig. 6:**
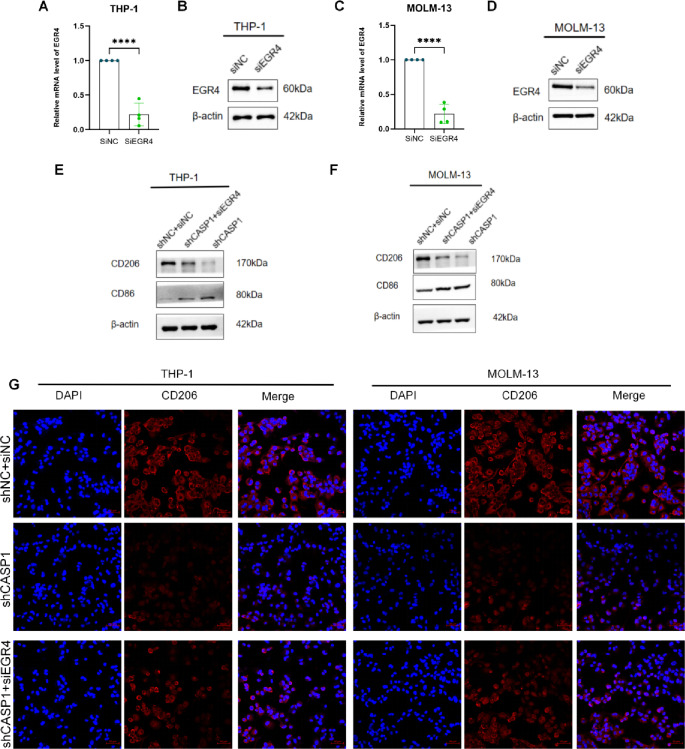

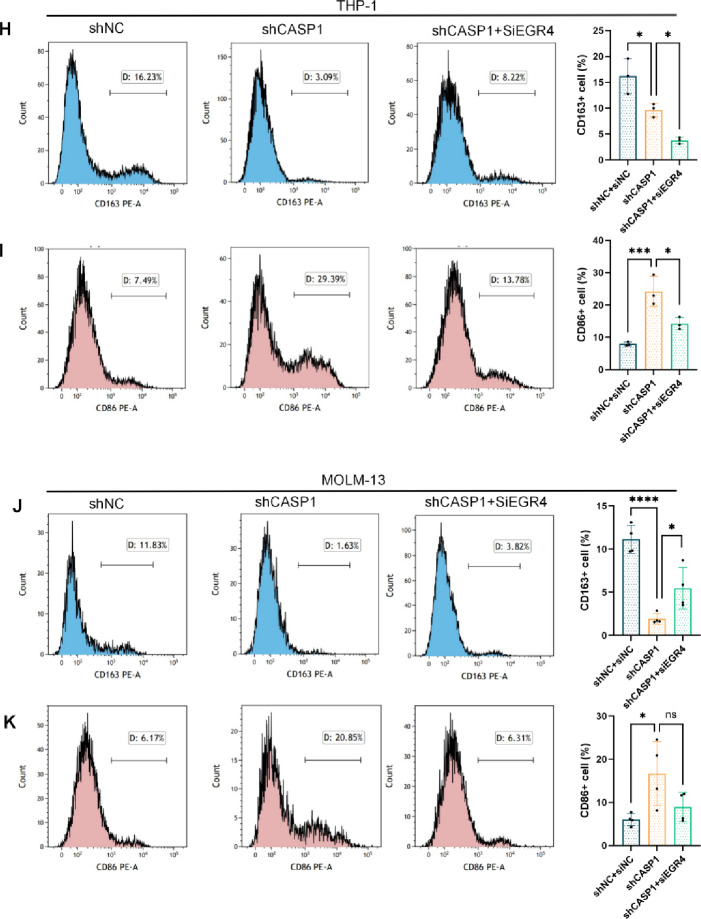
EGR4 KD rescues the macrophage polarization shift induced by CASP1 KD. (**A**,**C**) RT-qPCR analysis of EGR4 mRNA levels. (**B**,**D**) Representative immunoblot of EGR4 protein expression. (**E**,**F**) Representative immunoblots for CD206 and CD86 in macrophages following stimulation with CM from the indicated AML cell groups. (**G**) Representative immunofluorescence staining for CD206 (red) in macrophages.Nuclear staining was performed with DAPI (blue) (scale bar: 50 μm). (**H**–**K**) Flow cytometric analysis of (**H**,**J**) CD163^+^ and (**I**,**K**) CD86^+^ macrophage populations after treatment with CM from (**H**,**I**) THP-1 or (**J**,**K**) MOLM-13 models. Graphical summary shows representative histograms (left) and the corresponding quantification of positive cells (right), presented as mean ± SD (*n* ≥ 3). Cropped blot images are shown. Groupings of lanes from non-adjacent parts of the same gel are indicated by black borders. Full-length, uncropped original blots are provided in Supplementary Dataset.

Rescue experiments were performed to determine whether EGR4 is required for CASP1-mediated macrophage polarization. Efficient EGR4 KD was first verified in CASP1-KD AML cells (Fig. 6A–D).

At the single-cell level, flow cytometric quantification revealed that EGR4 KD significantly rescued the reduced CD163⁺ M2 macrophage population in both THP-1 and MOLM-13 models (Fig. 6H, J). Concurrently, it also significantly attenuated the CASP1 KD-induced increase in CD86⁺ M1 macrophages in THP-1 cells (Fig. 6I), while a similar decreasing trend was observed in the MOLM-13 model, albeit without reaching statistical significance (Fig. 6K).

This rescue effect was corroborated at the protein level. WB analysis demonstrated that the suppressed CD206 (M2-associated marker) expression in shCASP1 cells was partially restored upon EGR4 KD, while the enhanced CD86 (M1-associated marker) expression was markedly reduced (Fig. 6E, F). Immunofluorescence staining further confirmed the substantial restoration of CD206 fluorescence intensity and positive cell number in the shCASP1 + siEGR4 group compared to shCASP1 alone (Fig. 6G).

Collectively, these genetic rescue results establish EGR4 as an essential downstream effector through which CASP1 mediates the reprogramming of macrophage polarization, primarily by sustaining the M2 phenotype.

### The CASP1-EGR4 axis is linked to IL-10/p-STAT3 pathway activation during macrophage polarization

**Fig. 7:**
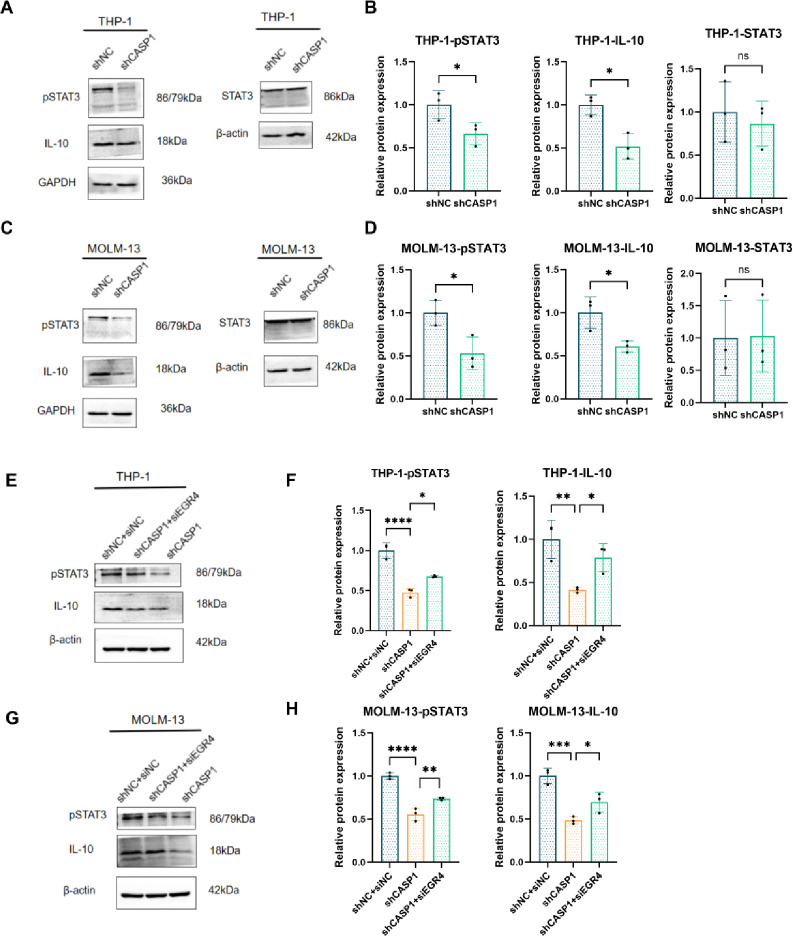
Mechanism of the CASP1-EGR4 axis through the IL-10/STAT3 pathway in macrophages. (**A**,**B**) Conditioned medium (CM) from THP-1 cells with CASP1 knockdown (KD) reduces the levels of IL-10 and phosphorylated STAT3 (p-STAT3), but not total STAT3, in recipient macrophages. (**A**) Representative western blots and (**B**) statistical summary. (**C**,**D**) CM from MOLM-13 cells with CASP1 KD reduces the levels of IL-10 and p-STAT3, but not total STAT3, in recipient macrophages. (**C**) Representative western blots and (**D**) statistical summary. (**E**,**F**) Additional KD of EGR4 in CASP1-KD THP-1 cells partially reverses the reduction of IL-10 and p-STAT3 in macrophages treated with the corresponding CM. (**E**) Representative western blots and (**F**) statistical summary. (**G**,**H**) Additional KD of EGR4 in CASP1-KD MOLM-13 cells partially reverses the reduction of IL-10 and p-STAT3 in macrophages treated with the corresponding CM. (**G**) Representative western blots and (**H**) statistical summary. Cropped blot images are shown. The STAT3 and p-STAT3 data are presented in separate frames to indicate they are from different blots. Full-length, uncropped original blots and, where informative, multiple exposures (e.g., for IL-10 and STAT3) are provided in Supplementary Dataset.

Having established EGR4 as a key downstream effector, we sought to identify the signaling pathways involved. Based on its established role as a master regulator of M2 polarization^23–25^, we focused on the IL-10/STAT3 pathway. Consistent with our hypothesis, CM from shCASP1 AML cells suppressed this pathway in both models, as shown by reduced levels of IL-10 and phosphorylated STAT3 (p-STAT3, but not total STAT3) compared to the shNC control (Fig. 7A–D). Crucially, concurrent KD of EGR4 in CASP1-KD cells (shCASP1 + siEGR4) significantly blocked this suppression, leading to a partial recovery of IL-10 and p-STAT3 levels (Fig. 7E–H). These genetic data functionally position the IL-10/p-STAT3 pathway as a critical downstream effector of the CASP1-EGR4 axis in regulating macrophage polarization.

### CASP1 knockdown suppresses AML progression and reprograms macrophages in vivo

**Fig. 8:**
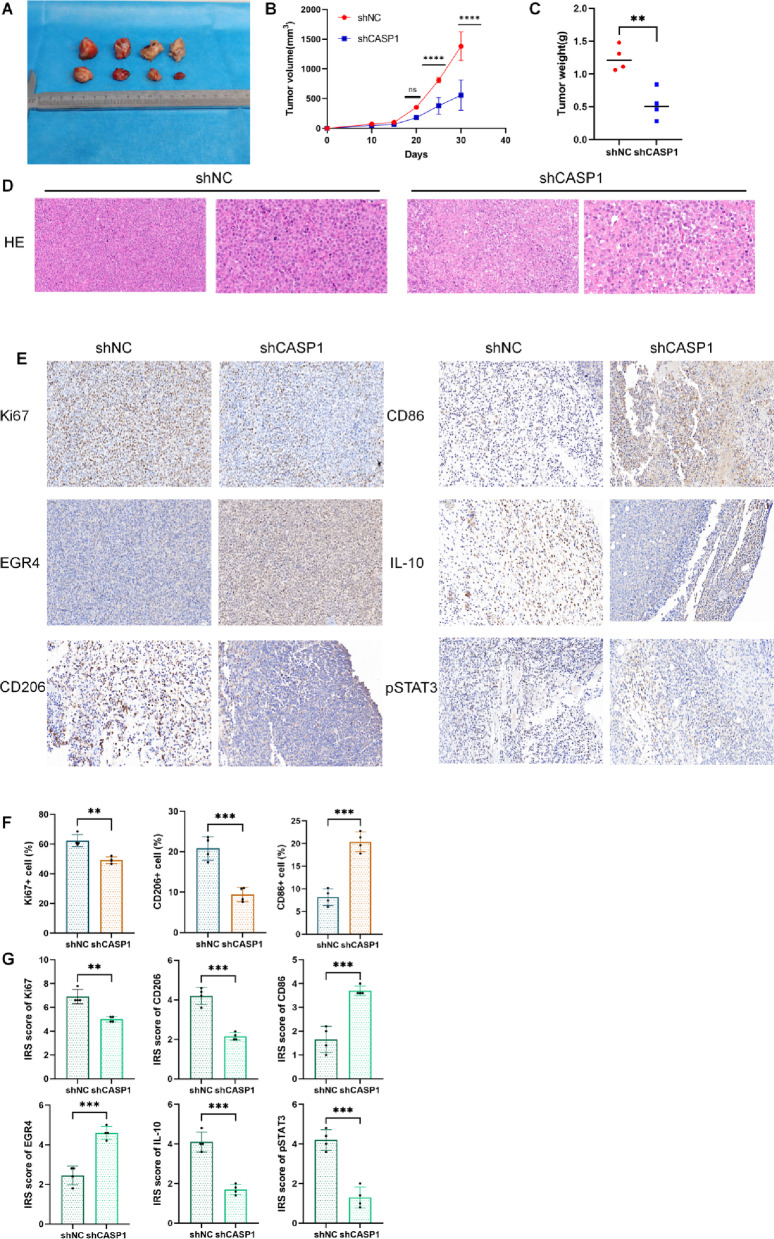
CASP1 KD suppresses AML tumor growth and reverses the immunosuppressive microenvironment in vivo. (**A**,**B**) Representative images of excised tumors (**A**) and tumor growth curves (**B**) from the shNC and shCASP1 groups. (**C**) Quantitative analysis of tumor weight from the respective groups. (**D**) Representative HE-stained tumor sections. Note the differences in cellularity and tissue organization. Scale bars: 50 μm (low magnification) and 20 μm (high magnification). (**E**) Representative immunohistochemical (IHC) images of tumor sections stained for the indicated markers (Ki67, EGR4, CD206, CD86, IL-10, p-STAT3). The images illustrate the corresponding cellular staining patterns and localization (nuclear: Ki67, EGR4, p-STAT3; membranous: CD206, CD86; cytoplasmic: IL-10). Visually, the shCASP1 group shows attenuated staining intensity for Ki67, CD206, IL-10, and p-STAT3, alongside enhanced staining for EGR4 and CD86, compared to the shNC control. (**F**–**G**) Dual-parameter quantification (positive cell percentage and IRS) for Ki-67, CD206, and CD86. Quantification for EGR4, IL-10, and p-STAT3 is presented as the IRS. Graphical data depict the mean ± SD. Significance (as determined by comparison to shNC group) is indicated as follows: **p < 0.01, ***p < 0.001.

An in vivo xenograft study was conducted by injecting NOD-SCID mice with MOLM-13 cells expressing shCASP1 or shNC to evaluate the effect of CASP1 KD on AML progression. Despite a sample size (*n* = 4 per group) that may limit insights into subtle effects, CASP1 KD still elicited a significant suppression of tumor growth, evidenced by markedly lower final tumor volume and weight (Fig. 8A–C). HE staining revealed morphological differences: tumors in the shCASP1 group exhibited reduced cellular density and a more organized architecture compared to the shNC control tumors (Fig. 8D). To investigate the underlying mechanisms of tumor suppression, we performed IHC analysis of the TME. This analysis revealed multi-faceted alterations upon CASP1 KD (Fig. 8E–G). Specifically, a marked decrease in tumor proliferative activity was observed, as determined by a reduction in both the Ki-67^+^ cell percentage and the IRS, with representative images showing a clear transition from frequent, strong nuclear staining in controls to sparse, weak staining. Consistent with our in vitro findings, EGR4 expression was significantly upregulated, reflected by enhanced nuclear staining intensity in tumor cells. Furthermore, assessment of polarization markers revealed that CASP1 KD skewed macrophages toward an M1 phenotype, manifesting as suppressed membrane staining for the M2-associated marker CD206 and elevated membrane staining for the M1-associated marker CD86 across both percentage and IRS evaluations. This repolarization was accompanied by a significant attenuation of the IL-10/p-STAT3 signaling axis, as indicated by lower IRS for both molecules, corresponding to visibly diminished cytoplasmic staining for IL-10 and nuclear staining for p-STAT3 within the TME.

Together, these in vivo results demonstrate that CASP1 KD in AML cells not only curbs tumor growth but also reprograms tumor-associated macrophages toward an M1-like phenotype, associated with EGR4 upregulation and suppression of IL-10/p-STAT3 signaling.

## Discussion

Beyond its well-established roles in inflammasome activation and pyroptosis^8,26^, our study unveils a novel, tumor cell-intrinsic function of CASP1 in acute myeloid leukemia (AML): fostering an immunosuppressive microenvironment. This discovery comes at a time of growing therapeutic interest in the leukemic niche, ranging from protective niche editing^27^ to modulating inflammatory immune cells^28^. Uniquely, we have identified the CASP1-EGR4 axis as a key mechanism through which AML cells actively reprogram their surrounding milieu. Clinically, CASP1 expression correlates with M2 macrophage infiltration, and mechanistically, we demonstrate that CASP1 in AML cells drives macrophage polarization towards a pro-tumor phenotype primarily by suppressing the transcription factor EGR4. Notably, in our experimental model, while CASP1 KD in AML cells did not potently induce the transcription of IL-1β—a canonical downstream effector—in recipient macrophages, it significantly suppressed the M2 polarization program. This suggests that the pro-tumor effect of AML-cell-intrinsic CASP1 is mediated largely through the regulation of EGR4 and the consequent alteration of a secretory factor profile distinct from the classical IL-1β-dominated inflammatory pathway.

EGR4 has documented roles in neuromodulation, muscle differentiation^29,30^, and context-dependent functions in other cancers^31–33^. For instance, it can promote colorectal cancer proliferation by activating the TNF-α/NF-κB pathway^32^ yet it also acts as a key target for gramicidin-mediated inhibition of cholangiocarcinoma growth^34^. In the immune context, it precisely regulates T cell function and Th1 differentiation and anti-tumor immunity through suppression via the calcium signaling pathway^35,36^. However, its role in AML remained unclear. Our data now establish EGR4 as a crucial tumor suppressor and innate immune signaling regulator in AML. We found that CASP1 is a potent negative regulator of EGR4, with CASP1 KD significantly upregulating EGR4 expression. Crucially, rescue experiments definitively showed that the anti-tumor and macrophage reprogramming effects resulting from CASP1 KD are critically dependent on the restoration of EGR4. Transcriptomic changes following EGR4 upregulation indicated a broad attenuation of pro-tumorigenic pathways.

We propose a model wherein therapeutic targeting of the CASP1-EGR4 axis counteracts immunosuppression via a defined signaling cascade: CASP1 KD relieves the suppression of EGR4, which subsequently acts as a molecular brake on the IL-10/p-STAT3 signaling pathway. This disruption primarily impedes the M2 polarization program, shifting macrophages toward a less immunosuppressive state, and undermines a key mechanism of immune escape in this context. The consistent reduction in canonical M2 markers (CD163, CD206) and the M2-sustaining IL-10/p-STAT3 pathway, alongside the induction of M1-associated CD86, strongly implies a functional re-education of TAMs toward a phenotype with potential anti-tumor capacities, a premise that invites direct functional investigation in future studies.

Our study has definitively established that the CASP1-EGR4 axis within AML cells is a key regulator of macrophage polarization toward an M2-like phenotype. The consequent remodeling of the immune microenvironment, observed both in vitro and in vivo, is strongly associated with impaired tumor growth in our xenograft model. This compelling association leads us to propose that the tumor-promoting function of AML-cell-intrinsic CASP1 is mediated primarily through this novel EGR4-dependent pathway. A critical and logical next step is to directly demonstrate how the CASP1-EGR4-reprogrammed macrophages mechanistically contribute to AML progression. The tumor-suppressive effect observed in immunodeficient mice specifically suggests the involvement of T-cell-independent mechanisms, such as providing pro-survival signals, metabolic support, or fostering a protective niche. Elucidating these specific downstream effector functions will be essential to fully understand the therapeutic implications of disrupting the CASP1-EGR4 axis.

The translational potential of this axis is robustly supported by our in vivo data. In xenograft models, CASP1 KD suppressed AML growth, accompanied with EGR4 upregulation, suppression of the IL-10/p-STAT3 pathway, and a shift in macrophage phenotype from M2 to M1-like, confirming the pathway’s role within the TME. This finding elevates the CASP1-EGR4 axis from a correlative signal to a potential therapeutic target with high translational value.

The availability of selective CASP1 inhibitors like VX-765 (Belnacasan)^37^, which has an established safety profile in humans and exhibits bioactivity in the hematopoietic system^38^, represents a viable therapeutic avenue for exploration. Our discovery of the CASP1-EGR4 axis, a novel regulator of the AML immune microenvironment, provides a mechanistic framework for re-evaluating the potential anti-tumor effects of such compounds. This finding suggests the hypothesis that in AML, beyond its canonical role in inhibiting pyroptosis, VX-765 might also confer benefit by targeting this axis to counteract pro-tumorigenic macrophage polarization. Future studies are necessary to directly test the impact of CASP1 inhibition on the CASP1-EGR4-IL-10/p-STAT3 pathway and validate this concept.

This study has certain limitations that define the scope of our current conclusions and highlight promising avenues for future investigation. (1) Model system considerations: Our findings are established in established AML cell lines and a subcutaneous xenograft model. The use of THP-1-derived macrophages, while a well-established and reproducible model for studying human macrophage polarization in vitro, may not fully capture the heterogeneity and ontogeny of TAMs within the complex AML bone marrow niche. While these systems were instrumental in defining the local CASP1-EGR4-macrophage axis, they do not fully capture the genetic heterogeneity of primary AML or the systemic disease context, including the bone marrow niche and a complete immune landscape. Future validation in patient-derived samples and systemic/immunocompetent animal models will be crucial to assess broad translational relevance. (2) Mechanistic elucidation: While we identify EGR4 as a key downstream effector, the precise molecular mechanism by which CASP1 regulates EGR4 expression (transcriptional vs. indirect) remains to be determined. Furthermore, the conditioned medium contains a complex mixture of factors; dissecting the specific secretory mediators (e.g., soluble proteins vs. exosomes) and clarifying the cellular source of key signals like IL-10 within the niche are important next steps for deeper mechanistic understanding. Additionally, while our study defines the axis regulating macrophage polarization states, direct assessment of the functional consequences (e.g., on phagocytosis or T cell activity) will be important to fully confirm the therapeutic potential of disrupting this pathway. (3) Experimental design for pathway mapping: Our rescue experiments demonstrate the necessity of EGR4 within this axis. A more detailed mapping of the pathway, including the contribution of basal EGR4 activity through an siEGR4-only control, would further refine the model and is a logical focus for subsequent studies.

## Conclusion

Our study elucidates a novel pro-tumorigenic signaling axis in AML cells, comprising the CASP1-EGR4-IL-10/p-STAT3 pathway. We demonstrate that upregulation of CASP1 expression in leukemic cells promotes macrophage polarization towards an M2 phenotype through suppression of EGR4—which we identify as a critical brake in this process. Collectively, our findings in experimental AML models establish the CASP1-EGR4 pathway as a compelling therapeutic target for inhibiting pro-tumorigenic M2 macrophage polarization.

## Supplementary Information

Below is the link to the electronic supplementary material.

Supplementary Material 1

Supplementary Material 2

## Data Availability

The data that support the findings of this study are available from the corresponding author upon reasonable request.
