# Supplementary material for: The Caspase-1-EGR4 axis regulates macrophage repolarization in acute myeloid leukemia cells
Source: Sci Rep. 2026 Feb 27;16:11319. doi: 10.1038/s41598-026-41381-x (PMC13049065; doi:10.1038/s41598-026-41381-x)
Supplement: Supplementary file 1 — Supplementary Material 1 [file 41598_2026_41381_MOESM1_ESM.docx]

Supplementary Material-Tables

****The Caspase-1-EGR4 axis drives** acute myeloid leukemia** ****progression by orchestrating macrophage repolarization****

Yi Qian^1,2^,Yue Chen^1^, Zu-Xi Feng^1^, Xiao-Feng Zhu^2^, Li Zhang^1^, Hao Xiong^1,2^, Xiang-hui Zhang^5^, Jun Bai^3^, Yan-hong Li^3^, Yu-xian Wang^3^, Lijuan Li^1,3,4*^, Liansheng Zhang^1,3,4*^

Lijuan Li, Department of Hematology, The Second Hospital and Clinical Medical School, Lanzhou University, Lanzhou City 730030, China. E-mail: lilijuan1232025@163.com.

Liansheng Zhang, Department of Hematology, The Second Hospital and Clinical Medical School, Lanzhou University, Lanzhou City 730030, China. E-mail: doctorzhanglsh@sina.com.

This file includes: Supplementary Table S1- S2.

****Supplementary Table S1.**** Primer sequences for qPCR analysis of macrophage polarization marker genes**.**

| **primers** | **sequences** |
| --- | --- |
| β-actin Forward | 5′CCTGTACGCCAACACAGTGC3′ |
| β-actin Reverse | 3′ATACTCCTGCTTGCTGATCC5′ |
| IL-10 Forward | 5′GACTTTAAGGGTTACCTGGGTTG3′ |
| IL-10 Reverse | 3′TCACATGCGCCTTGATGTCTG5′ |
| IL-4 Forward | 5′CCAACTGCTTCCCCCTCTG3′ |
| IL-4 Reverse | 3′TCTGTTACGGTCAACTCGGTG5′ |
| TGF-β Forward | 5′GGCCAGATCCTGTCCAAGC3′ |
| TGF-β Reverse | 3′GTGGGTTTCCACCATTAGCAC5′ |
| Arg1 Forward | 5′CTGCTCATCTATACACGGTTACC3′ |
| Arg1 Reverse | 3′CCAGTCCGTCAACATCAAAACT5′ |
| IL-6 Forward | 5′ACTCACCTCTTCAGAACGAATTG3′ |
| IL-6 Reverse | 3′CCATCTTTGGAAGGTTCAGGTTG5′ |
| IL-1β Forward | 5′ATGATGGCTTATTACAGTGGCAA3′ |
| IL-1β Reverse | 3′GTCGGAGATTCGTAGCTGGA5′ |
| IL-12 Forward | 5′CCTTGCACTTCTGAAGAGATTGA3′ |
| IL-12 Reverse | 3′ACAGGGCCATCATAAAAGAGGT5′ |
| TNF-α Forward | 5′CCTCTCTCTAATCAGCCCTCTG3′ |
| TNF-α Reverse | 3′GAGGACCTGGGAGTAGATGAG5′ |

**Supplementary Table S2. Antibody specifications. **All antibodies used in this study are specific for human antigens.****

| **antibodies** | **Host Species** | **catalog number** | **vendor** |
| --- | --- | --- | --- |
| anti-CASP1 | Rabbit | ab207802 | abcam,England |
| anti-EGR4 | Rabbit | ab198197 | abcam,England |
| anti- CD206 | Rabbit | MA5-32498 | Invitrogen, USA |
| anti- CD86 | Rabbit | SJ20-00 | HUABIO, China |
| anti- STAT3 | Rabbit | SY24-08 | HUABIO, China |
| anti- pSTAT3 | Rabbit | SZ43-01-08 | HUABIO, China |
| anti- Ki67 | Rabbit | SR00-02 | HUABIO, China |
| anti- EGR4 | Rabbit | HA500445 | HUABIO, China |
| anti- IL-10 | Rabbit | JE59-71 | HUABIO, China |
| anti-β- actin | Rabbit | 6609-1-lg | HUABIO, China |
| anti-GAPDH | Mouse | 60004-1-lg | Proteintech, China |
| Goat anti-Mouse | Mouse | SA00001-1 | Proteintech, China |
| Goat anti-Rabb | Rabbit | SA00001-2 | Proteintech, China |
| anti-human CD163-PE | Mouse | 12-1639-42 | Invitrogen, USA |
| anti-human CD86-PE | Mouse | 12-0869-42 | Invitrogen, USA |
