# Supplementary material for: The Caspase-1-EGR4 axis regulates macrophage repolarization in acute myeloid leukemia cells
Source: Sci Rep. 2026 Feb 27;16:11319. doi: 10.1038/s41598-026-41381-x (PMC13049065; doi:10.1038/s41598-026-41381-x)
Supplement: Supplementary file 2 — Supplementary Material 2 [file 41598_2026_41381_MOESM2_ESM.pdf]

Supplementary Dataset-Original Western Blot Images

The Caspase-1-EGR4 axis drives acute myeloid leukemia progression by orchestrating macrophage repolarization

Yi Qian[1,2],Yue Chen[1], Zu-Xi Feng[1], Xiao-Feng Zhu[2], Li Zhang[1], Hao Xiong[1,2], Xiang-hui Zhang[5], Jun Bai[3], Yan-hong Li[3], Yu-xian Wang[3], Lijuan Li[1,3,4*], Liansheng Zhang[1,3,4*]

Lijuan Li, Department of Hematology, The Second Hospital and Clinical Medical School, Lanzhou University, Lanzhou City 730030, China. E-mail: lilijuan1232025@163.com.

Liansheng Zhang, Department of Hematology, The Second Hospital and Clinical Medical School, Lanzhou University, Lanzhou City 730030, China. E-mail: doctorzhanglsh@sina.com.

This file includes: Original gels.

Figure1C

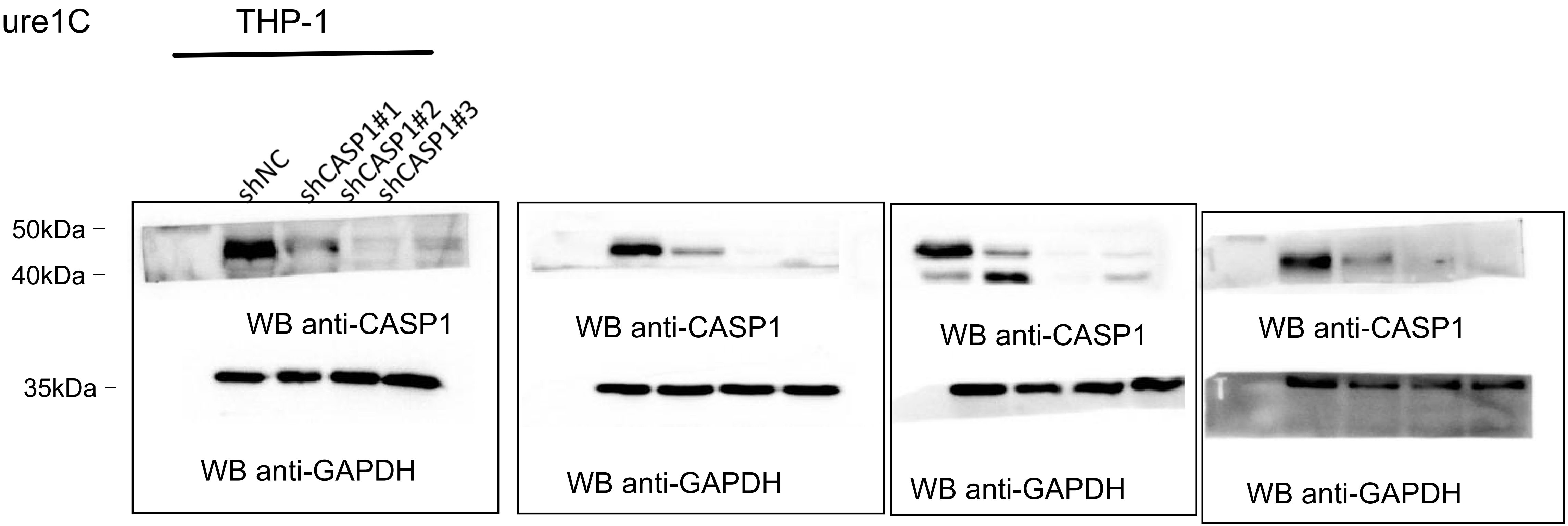
THP-1
shNC
shCASP1#1
shCASP1#2
shCASP1#3
50kDa
40kDa
35kDa
WB anti-CASP1
WB anti-GAPDH
WB anti-CASP1
WB anti-GAPDH
WB anti-CASP1
WB anti-GAPDH
WB anti-CASP1
WB anti-GAPDH

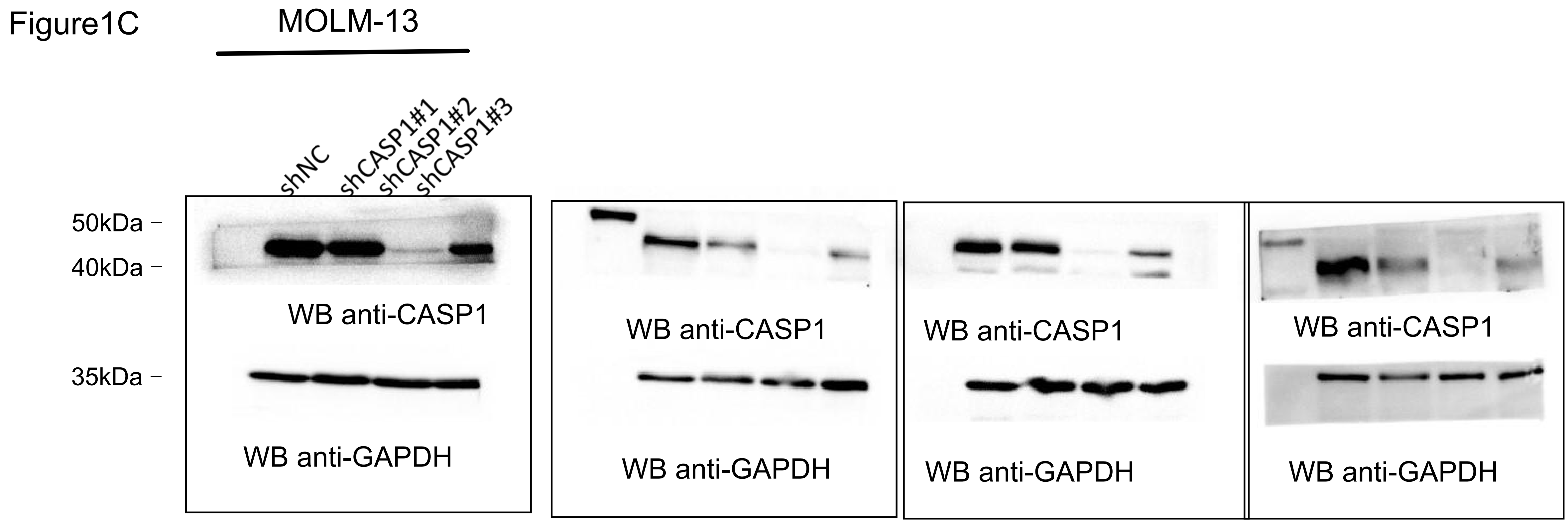
Figure1C
MOLM-13
shNC
shCASP1#1
shCASP1#2
shCASP1#3
50kDa
40kDa
35kDa
WB anti-CASP1
WB anti-GAPDH
WB anti-CASP1
WB anti-GAPDH
WB anti-CASP1
WB anti-GAPDH
WB anti-CASP1
WB anti-GAPDH

Figure 3A

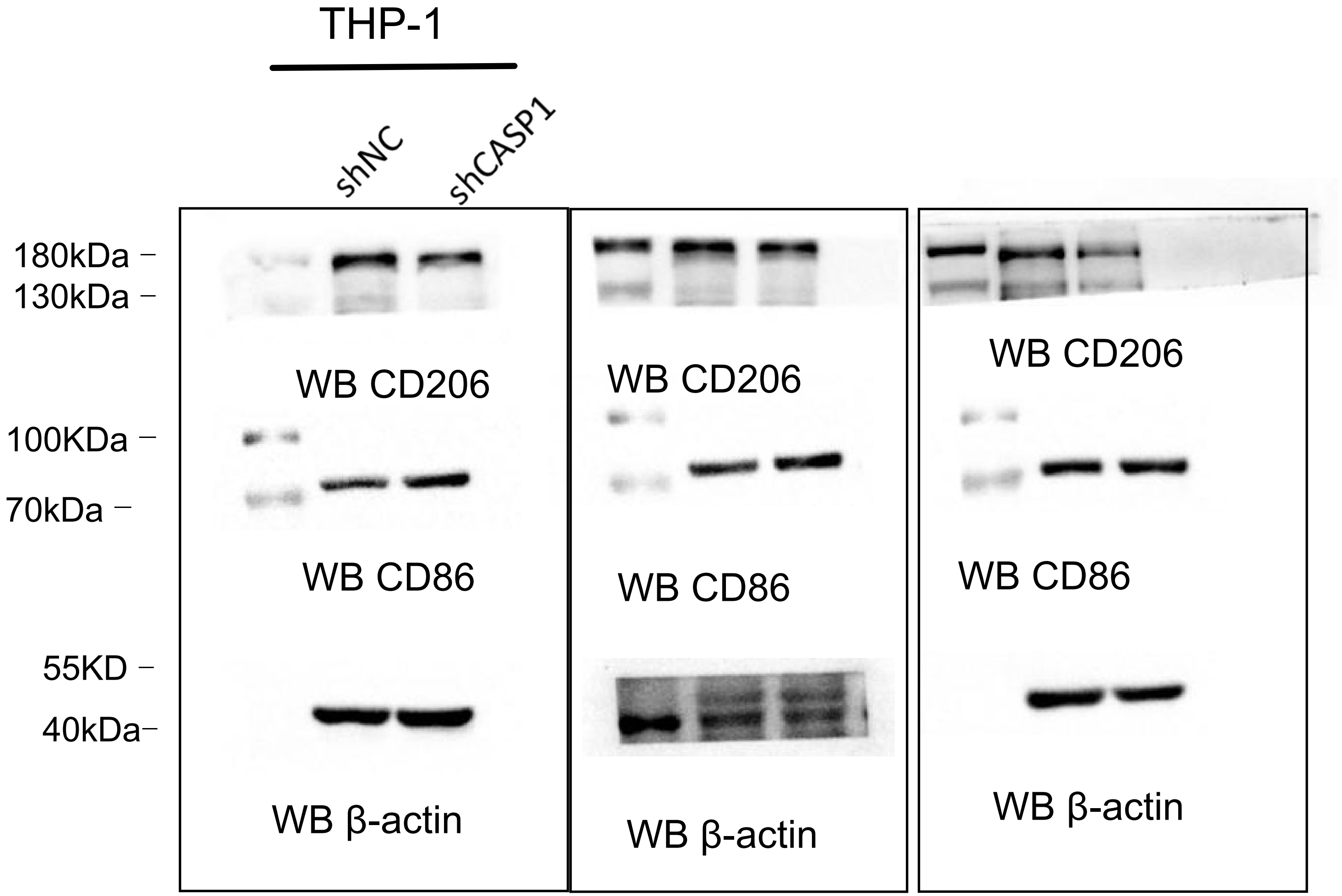
THP-1
shNC
shCASP1
180kDa
130kDa
WB CD206
100KDa
70kDa
WB CD86
55KD
40kDa
WB β-actin
WB CD206
WB CD86
WB β-actin
WB CD206
WB CD86
WB β-actin

Figure 3B

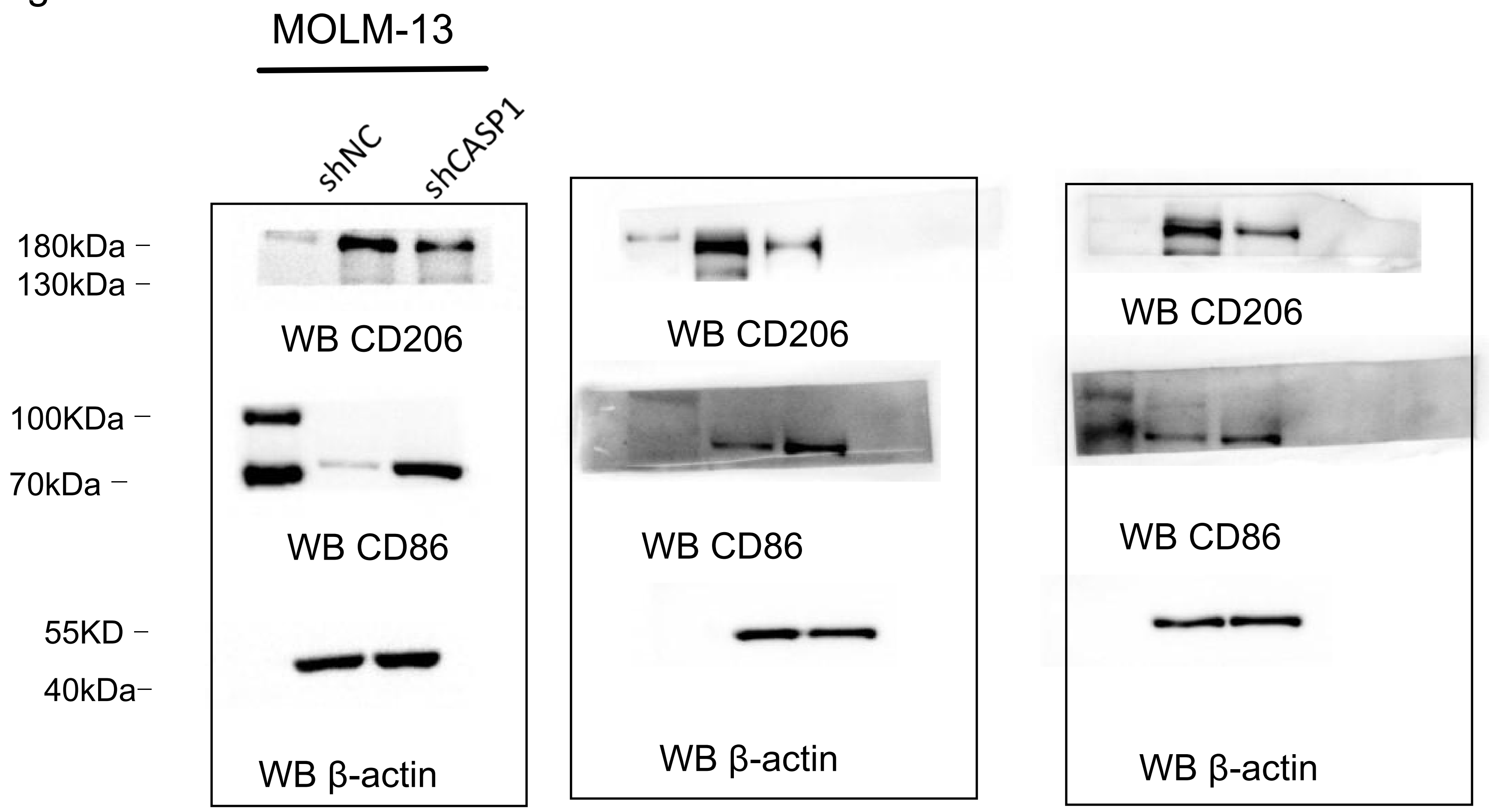
MOLM-13
shNC
shCASP1
180kDa
130kDa
WB CD206
100KDa
70kDa
WB CD86
55KD
40kDa
WB β-actin
WB CD206
WB CD86
WB β-actin
WB CD206
WB CD86
WB β-actin

Figure 5F

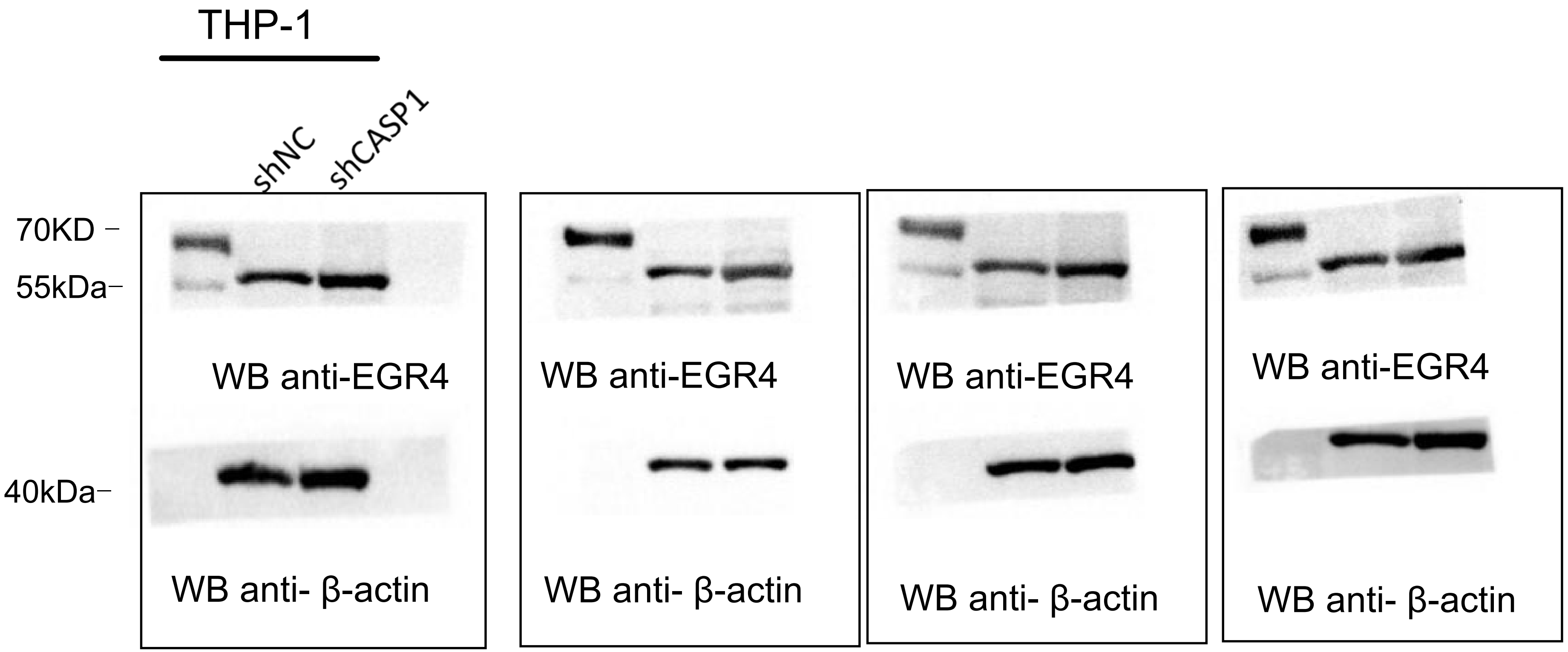
THP-1
shNC
shCASP1
70KD
55kDa
40kDa
WB anti-EGR4
WB anti- β-actin
WB anti-EGR4
WB anti- β-actin
WB anti-EGR4
WB anti- β-actin
WB anti-EGR4
WB anti- β-actin

Figure 5G

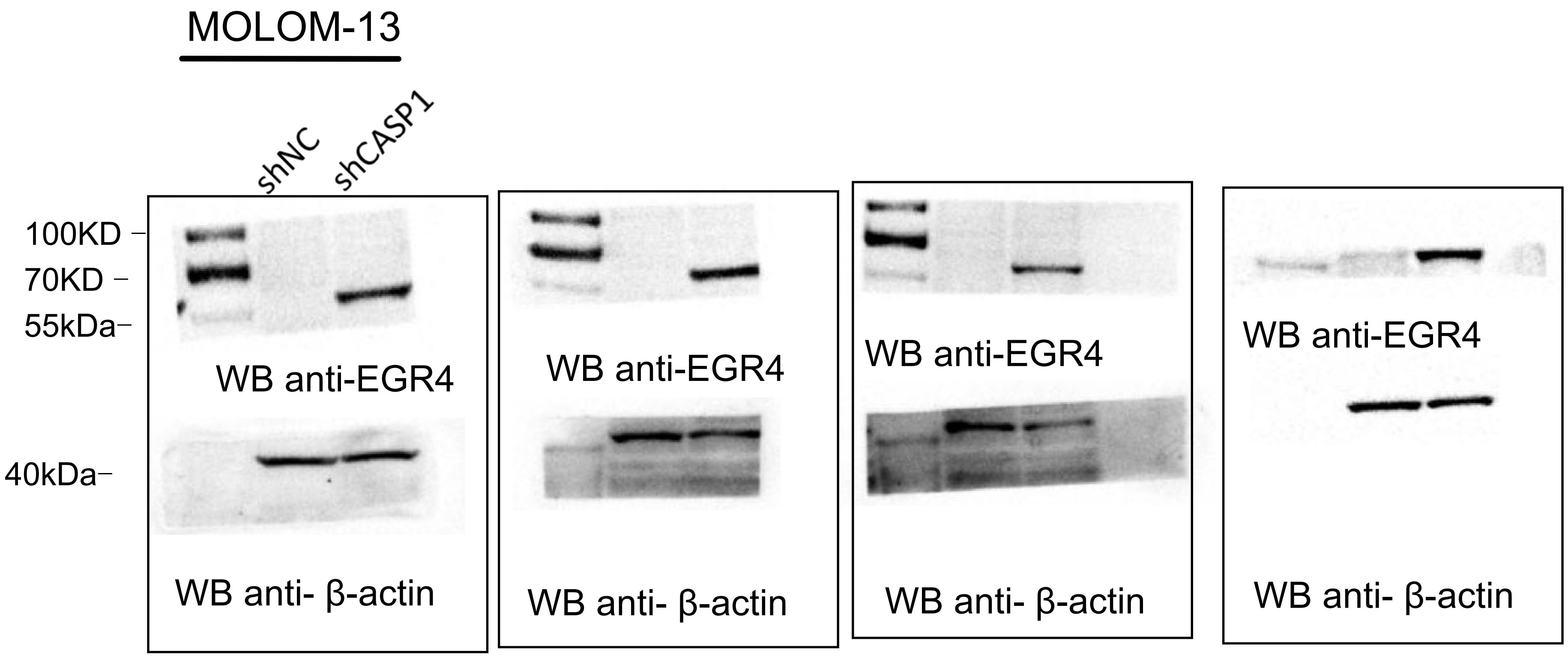

MOLOM-13
shNC
shCASP1
100KD
70KD
55kDa
40kDa
WB anti-EGR4
WB anti- β-actin
WB anti-EGR4
WB anti- β-actin
WB anti-EGR4
WB anti- β-actin
WB anti-EGR4
WB anti- β-actin

Figure 6B

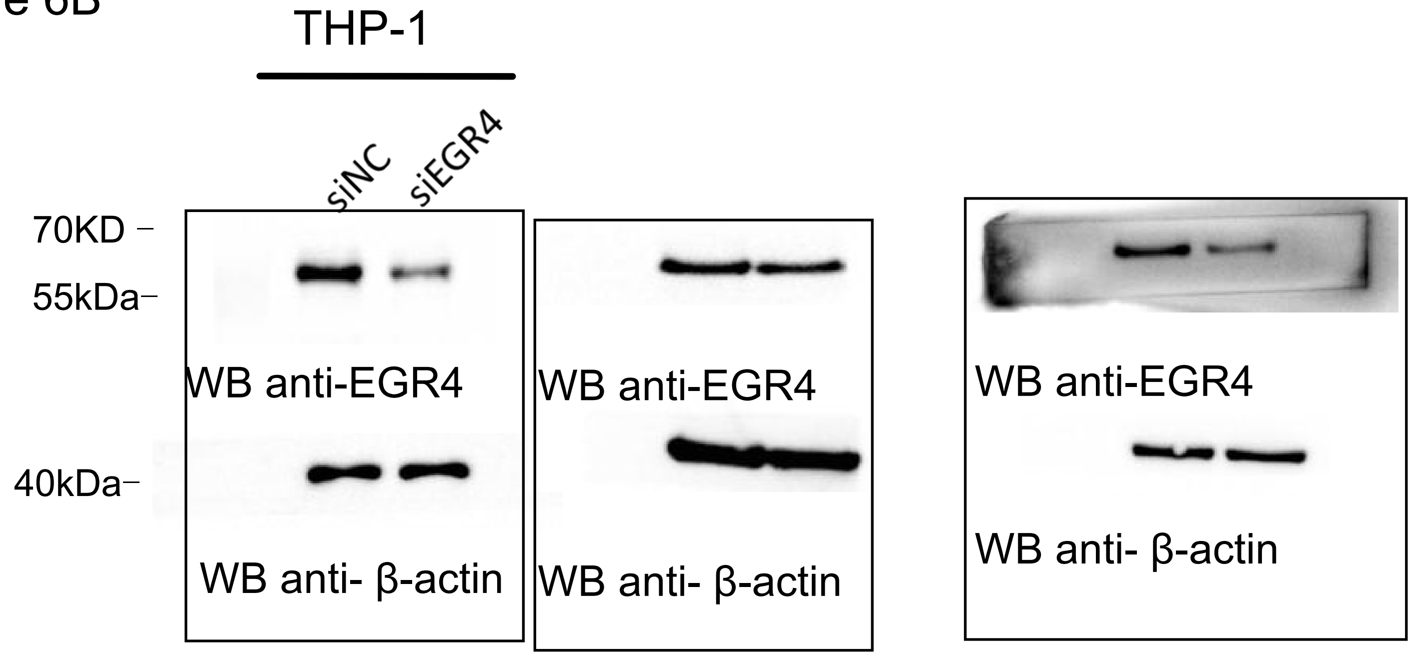

THP-1
siNC
siEGR4
70KD
55kDa
40kDa
WB anti-EGR4
WB anti- β-actin
WB anti-EGR4
WB anti- β-actin
WB anti-EGR4
WB anti- β-actin

Figure 6D

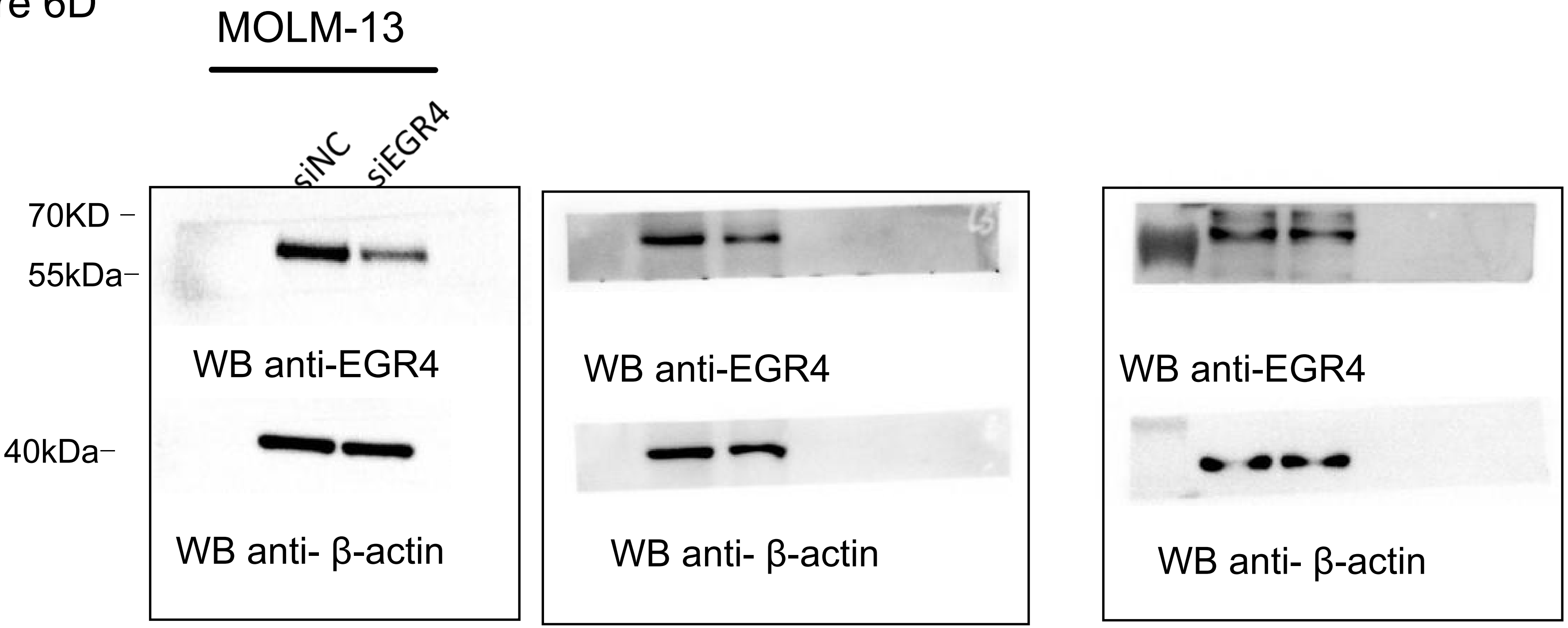
MOLM-13
siNC
siEGR4
70KD
55kDa
40kDa
WB anti-EGR4
WB anti- β-actin
WB anti-EGR4
WB anti- β-actin
WB anti-EGR4
WB anti- β-actin

Figure 6E

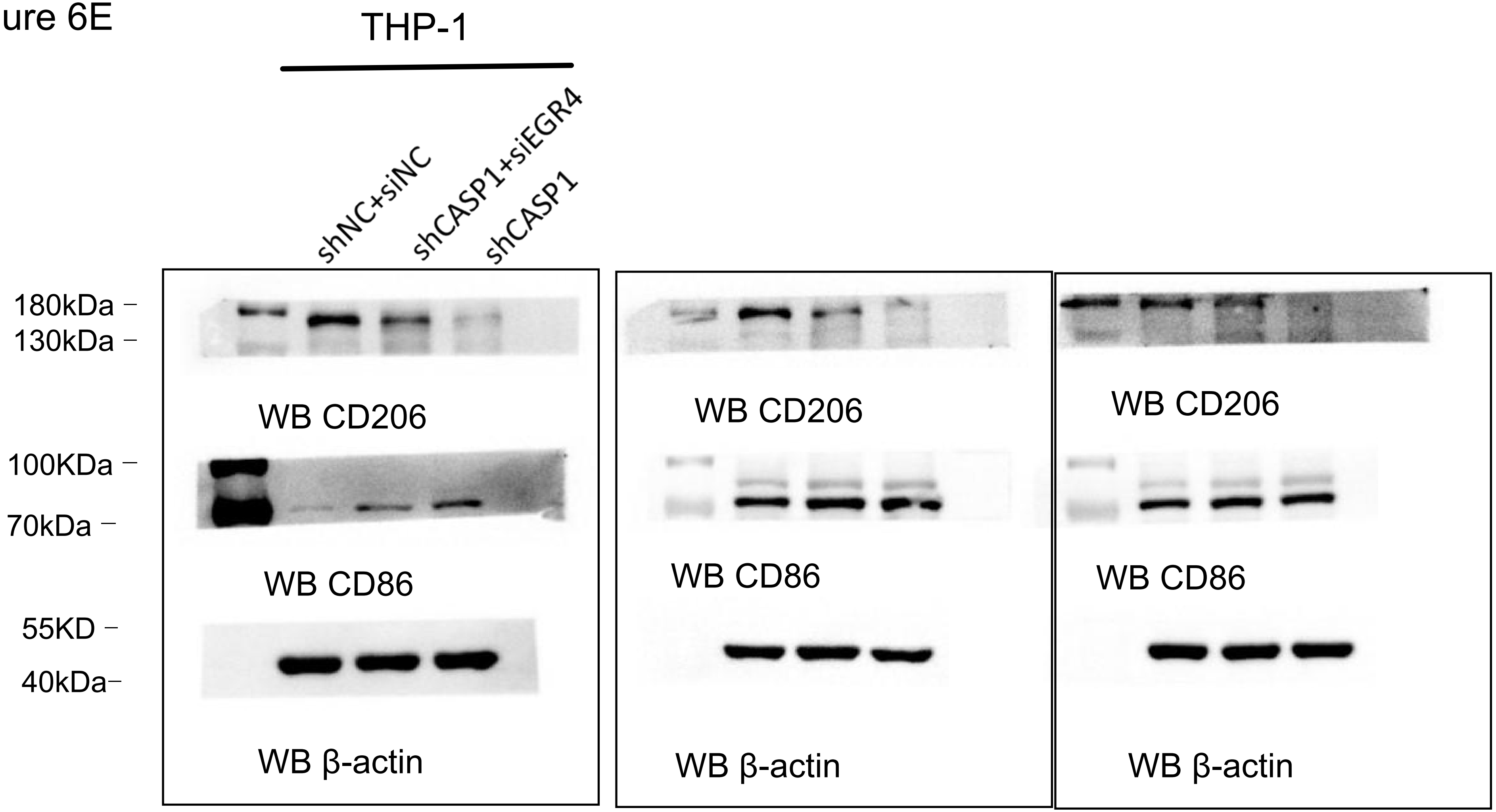
THP-1
shNC+siNC
shCASP1+siEGR4
shCASP1
180kDa
130kDa
WB CD206
100KDa
70kDa
WB CD86
55KD
40kDa
WB β-actin
WB CD206
WB CD86
WB β-actin
WB CD206
WB CD86
WB β-actin

Figure 6F

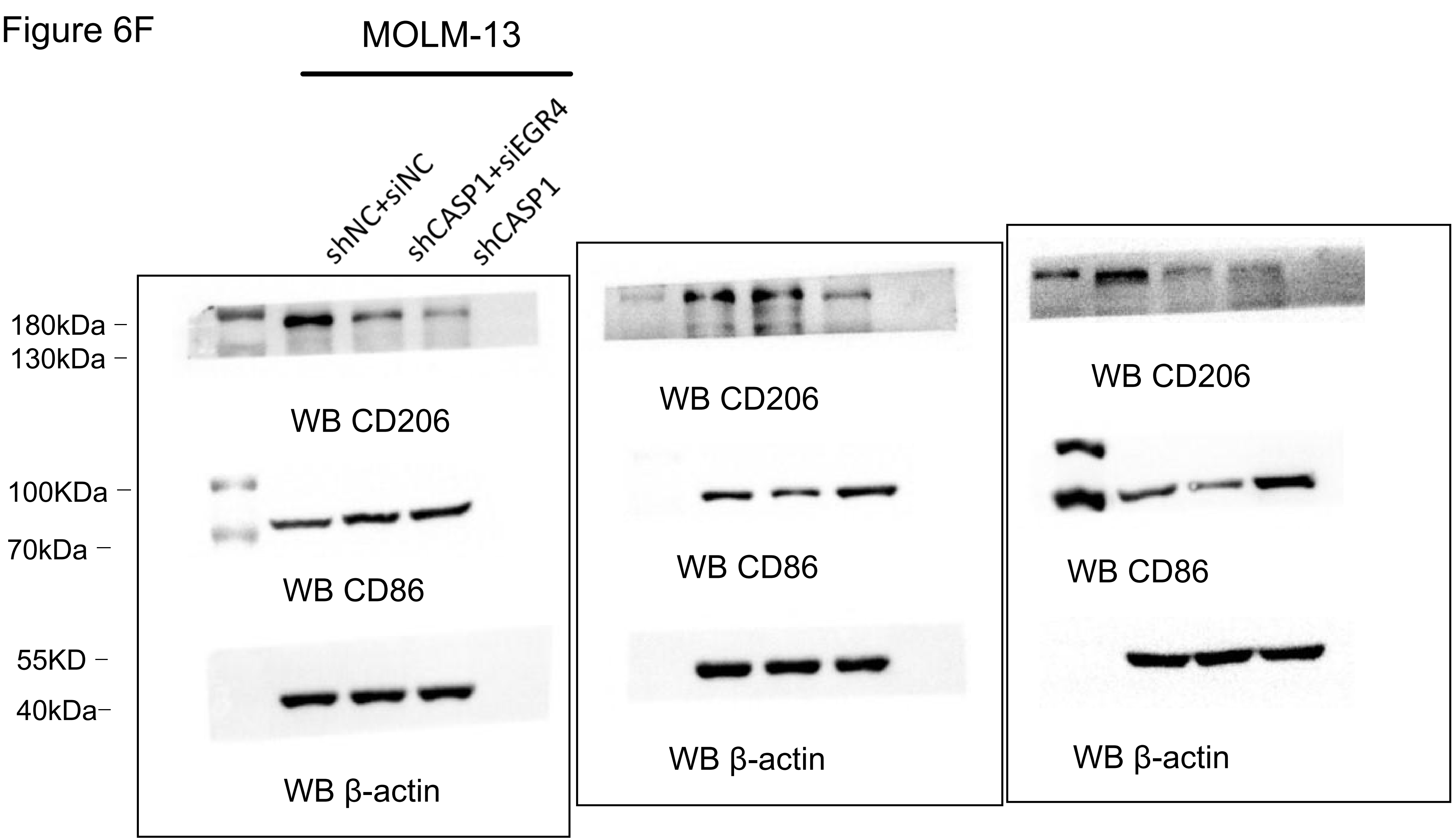

MOLM-13
shNC+siNC
shCASP1+siEGR4
shCASP1
180kDa
130kDa
WB CD206
100KDa
70kDa
WB CD86
55KD
40kDa
WB β-actin
WB CD206
WB CD86
WB β-actin
WB CD206
WB CD86
WB β-actin

Figure 7A

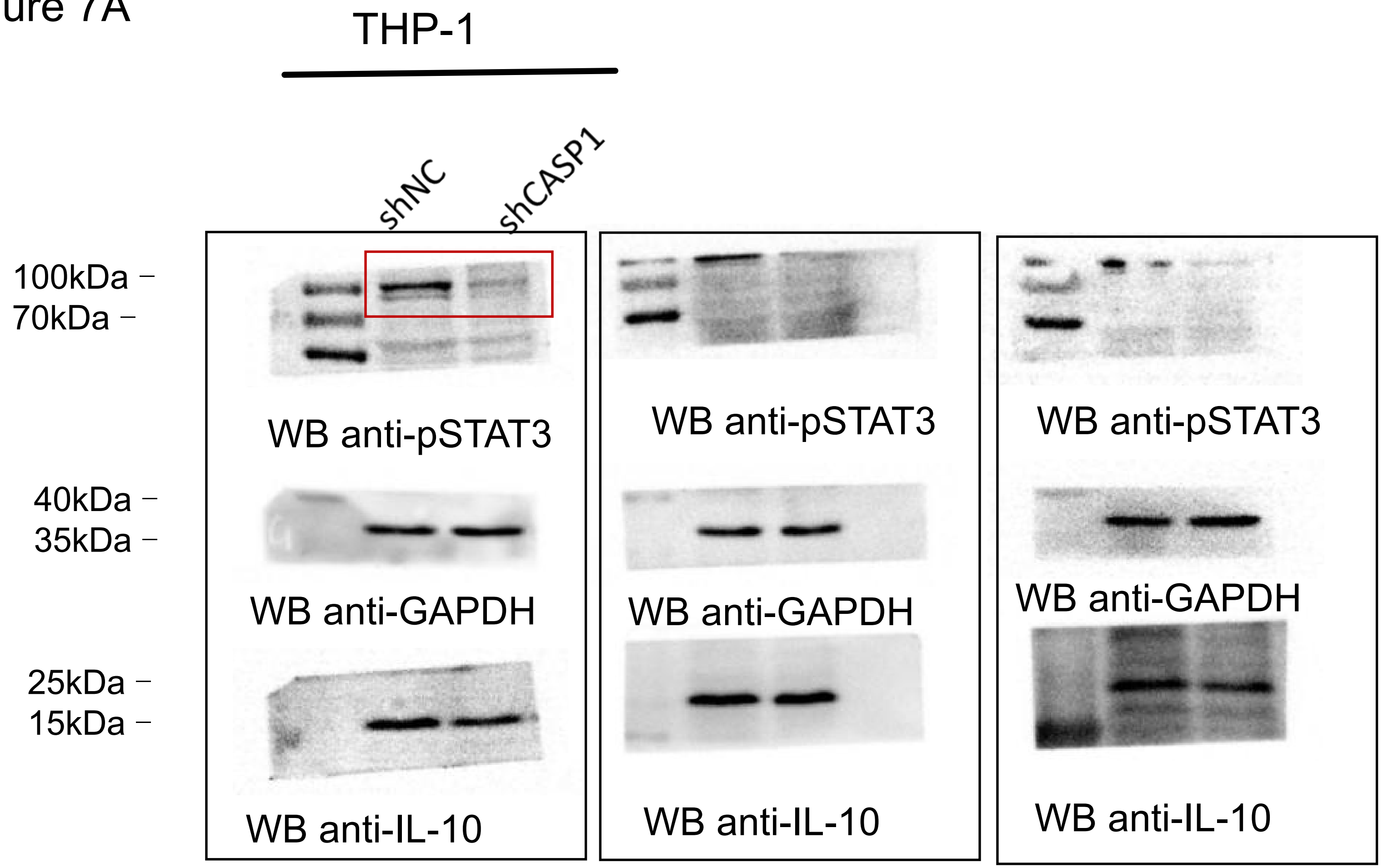
THP-1
shNC
shCASP1
100kDa
70kDa
WB anti-pSTAT3
40kDa
35kDa
WB anti-GAPDH
25kDa
15kDa
WB anti-IL-10
WB anti-pSTAT3
WB anti-GAPDH
WB anti-IL-10
WB anti-pSTAT3
WB anti-GAPDH
WB anti-IL-10

Figure 7A

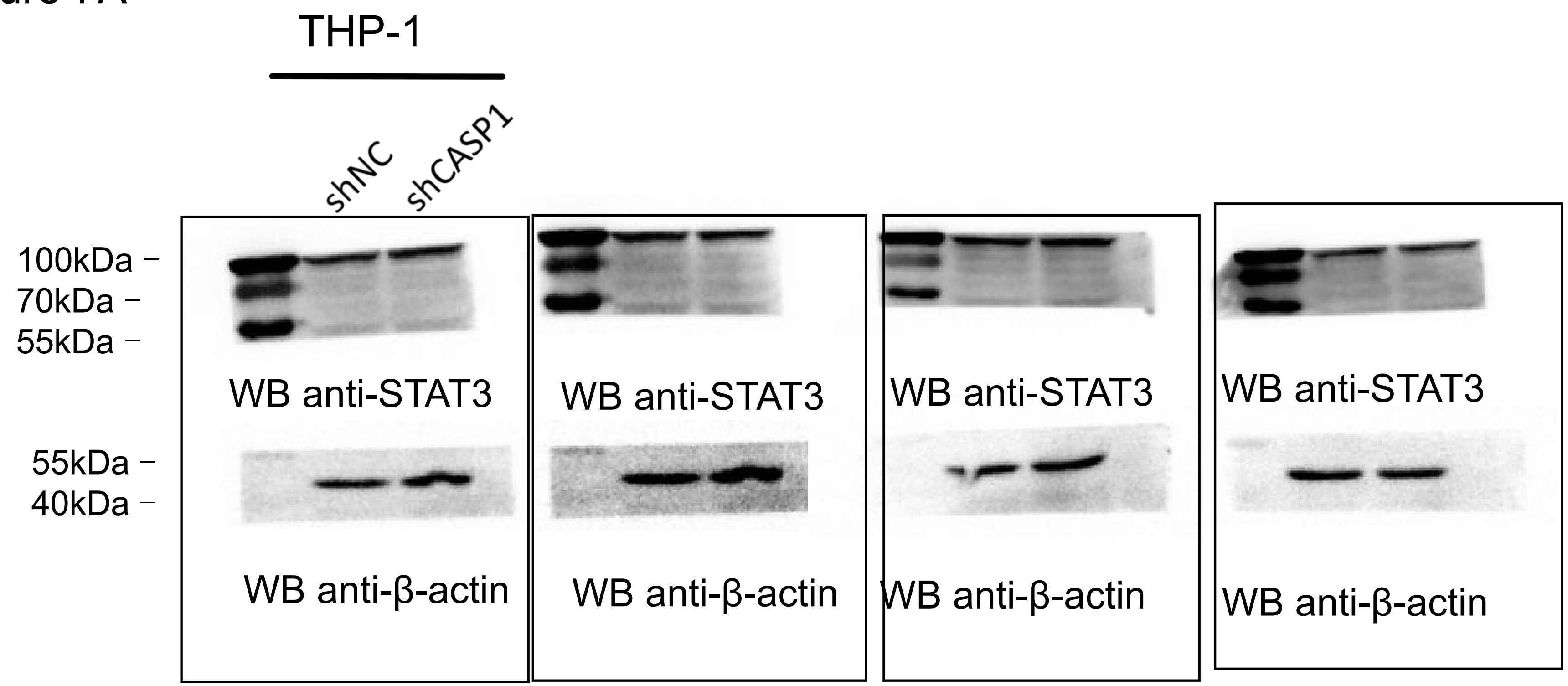

THP-1
shNC
shCASP1
100kDa
70kDa
55kDa
WB anti-STAT3
WB anti-STAT3
WB anti-STAT3
WB anti-STAT3
55kDa
40kDa
WB anti-β-actin
WB anti-β-actin
WB anti-β-actin
WB anti-β-actin

Figure 7B

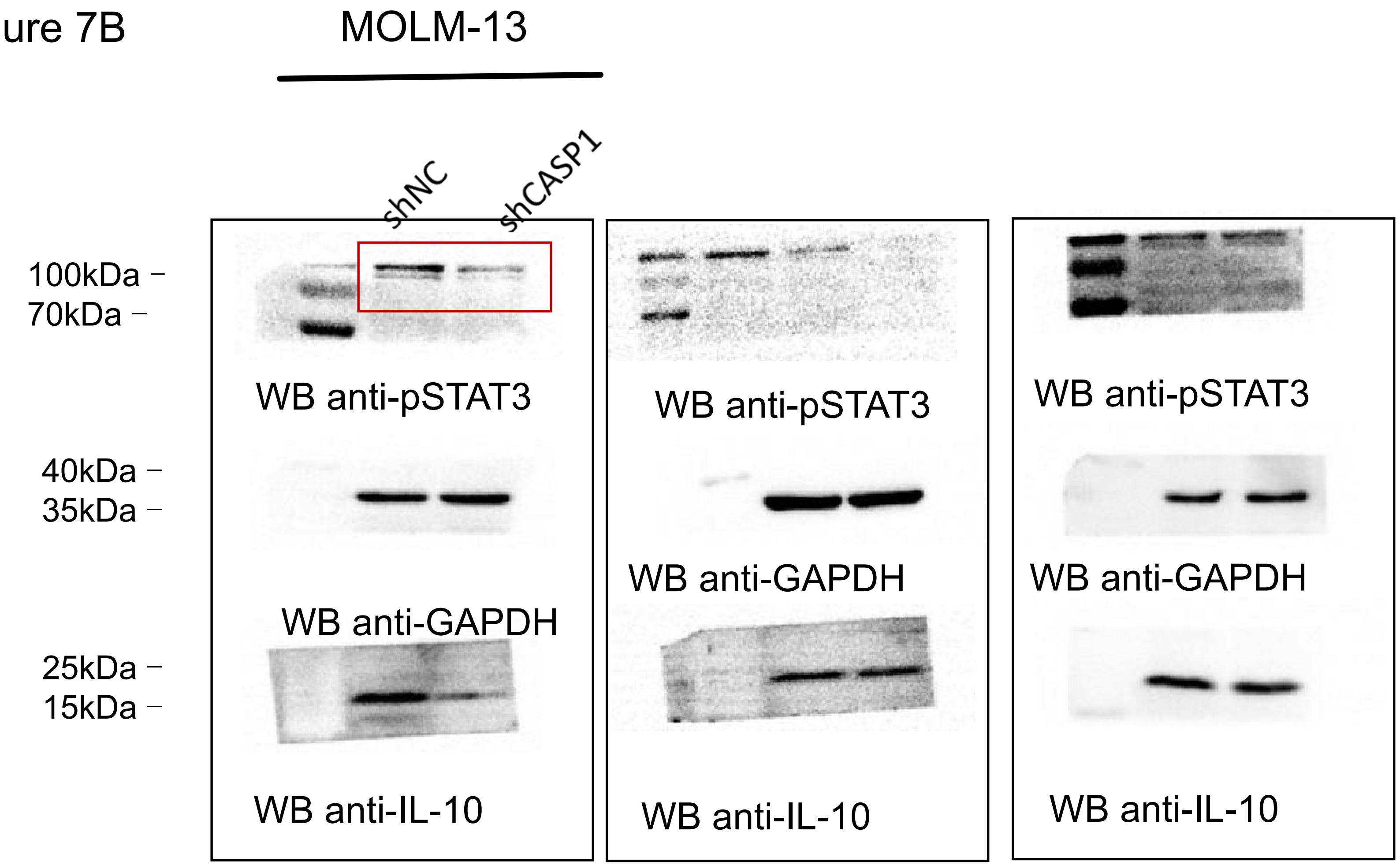
MOLM-13
shNC
shCASP1
100kDa
70kDa
WB anti-pSTAT3
40kDa
35kDa
WB anti-GAPDH
25kDa
15kDa
WB anti-IL-10
WB anti-pSTAT3
WB anti-GAPDH
WB anti-IL-10
WB anti-pSTAT3
WB anti-GAPDH
WB anti-IL-10

Figure 7B

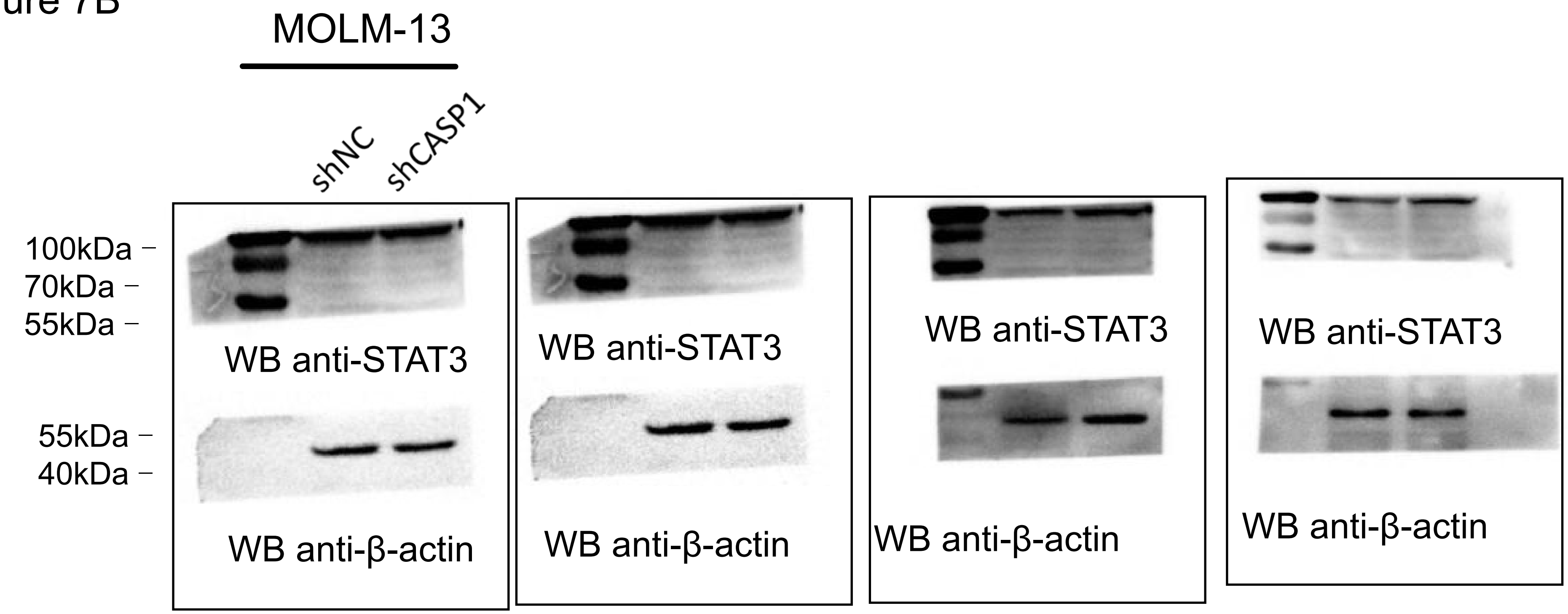
MOLM-13
shNC
shCASP1
100kDa
70kDa
55kDa
WB anti-STAT3
55kDa
40kDa
WB anti-β-actin
WB anti-STAT3
WB anti-β-actin
WB anti-STAT3
WB anti-β-actin
WB anti-STAT3
WB anti-β-actin

Figure 7C

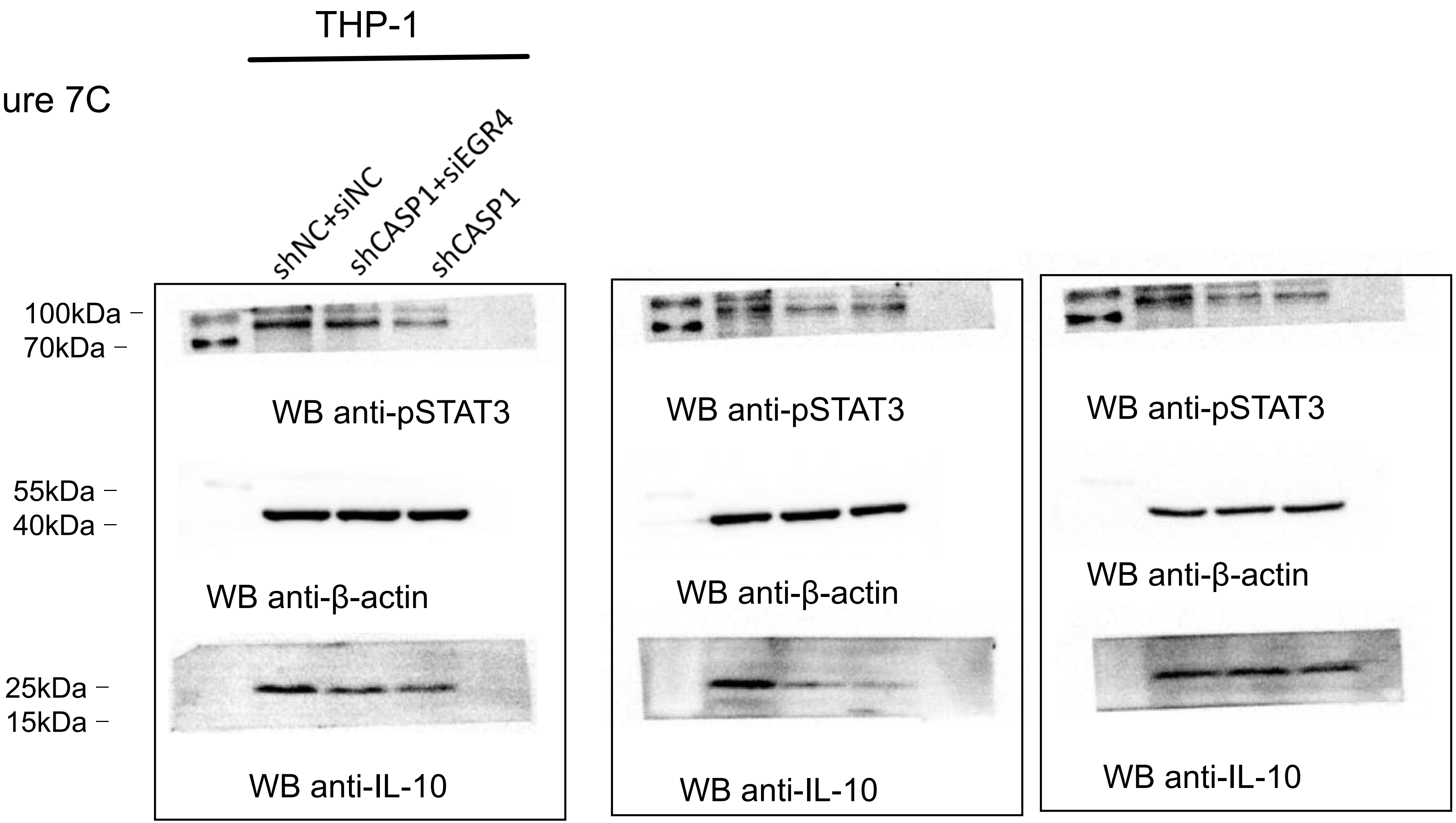
THP-1
shNC+siNC
shCASP1+siEGR4
shCASP1
100kDa
70kDa
WB anti-pSTAT3
55kDa
40kDa
WB anti-β-actin
25kDa
15kDa
WB anti-IL-10
WB anti-pSTAT3
WB anti-β-actin
WB anti-IL-10
WB anti-pSTAT3
WB anti-β-actin
WB anti-IL-10

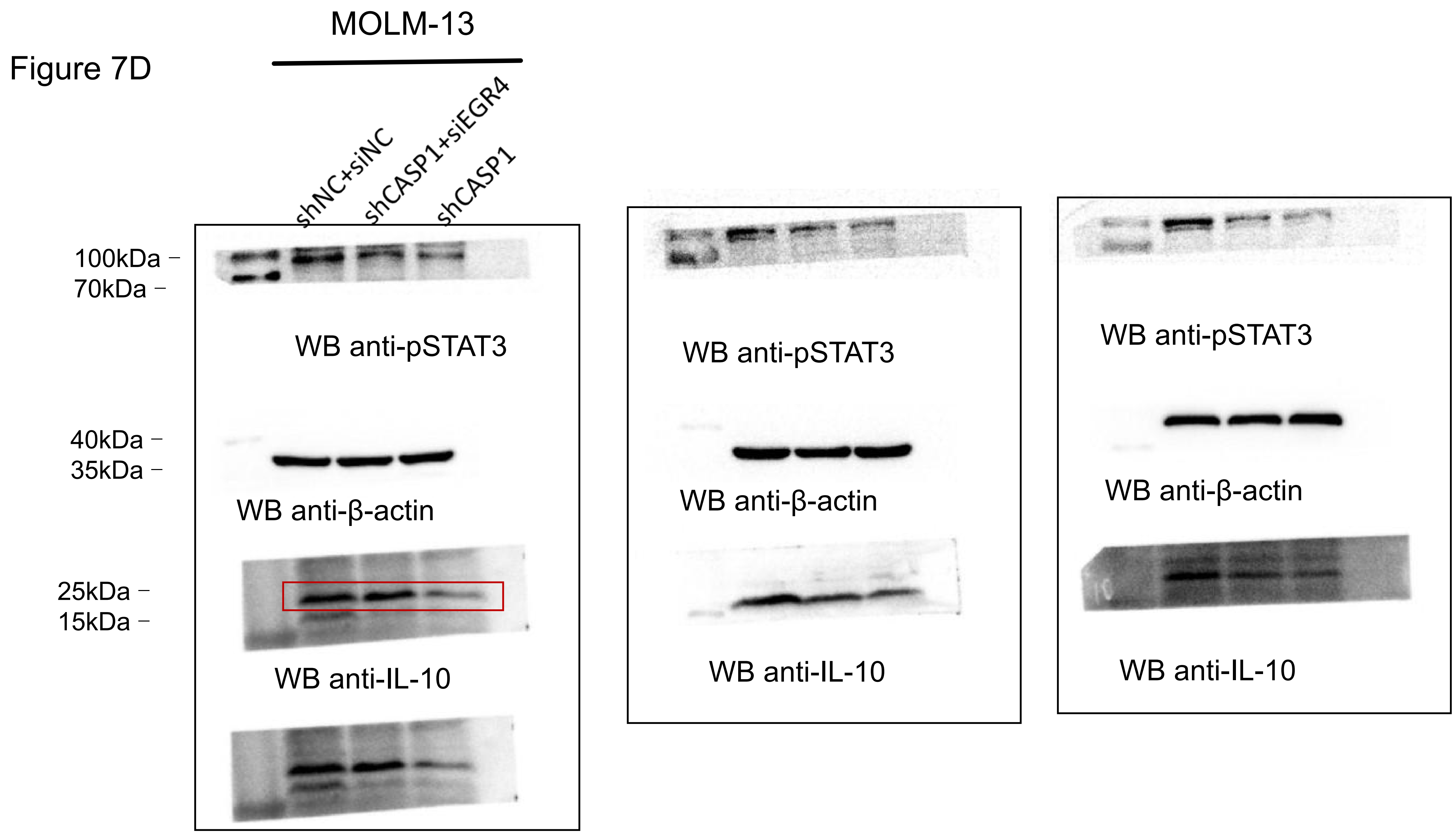
Figure 7D
MOLM-13
shNC+siNC
shCASP1+siEGR4
shCASP1
100kDa
70kDa
WB anti-pSTAT3
40kDa
35kDa
WB anti-β-actin
25kDa
15kDa
WB anti-IL-10
WB anti-pSTAT3
WB anti-β-actin
WB anti-IL-10
WB anti-pSTAT3
WB anti-β-actin
WB anti-IL-10
